# Changes in Tissue Fluidity Predict Tumor Aggressiveness In Vivo

**DOI:** 10.1002/advs.202303523

**Published:** 2023-08-08

**Authors:** Frank Sauer, Steffen Grosser, Mehrgan Shahryari, Alexander Hayn, Jing Guo, Jürgen Braun, Susanne Briest, Benjamin Wolf, Bahriye Aktas, Lars‐Christian Horn, Ingolf Sack, Josef A. Käs

**Affiliations:** ^1^ Soft Matter Physics Division Peter‐Debye‐Institute for Soft Matter Physics 04103 Leipzig Germany; ^2^ Institute for Bioengineering of Catalonia The Barcelona Institute for Science and Technology (BIST) Barcelona 08028 Spain; ^3^ Department of Radiology Charité‐Universitätsmedizin 10117 Berlin Germany; ^4^ Department of Hepatology Leipzig University Hospital 04103 Leipzig Germany; ^5^ Institute of Medical Informatics Charité‐Universitätsmedizin 10117 Berlin Germany; ^6^ Department of Gynecology Leipzig University Hospital 04103 Leipzig Germany; ^7^ Division of Breast, Urogenital and Perinatal Pathology Leipzig University Hospital 04103 Leipzig Germany

**Keywords:** cancer, in vivo magnetic resonance elastography, medical imaging, tissue fluidity, tumor mechanics

## Abstract

Cancer progression is caused by genetic changes and associated with various alterations in cell properties, which also affect a tumor's mechanical state. While an increased *stiffness* has been well known for long for solid tumors, it has limited prognostic power. It is hypothesized that cancer progression is accompanied by tissue *fluidization*, where portions of the tissue can change position across different length scales. Supported by tabletop magnetic resonance elastography (MRE) on stroma mimicking collagen gels and microscopic analysis of live cells inside patient derived tumor explants, an overview is provided of how cancer associated mechanisms, including cellular unjamming, proliferation, microenvironment composition, and remodeling can alter a tissue's *fluidity* and *stiffness*. In vivo, state‐of‐the‐art multifrequency MRE can distinguish tumors from their surrounding host tissue by their rheological fingerprints. Most importantly, a meta‐analysis on the currently available clinical studies is conducted and universal trends are identified. The results and conclusions are condensed into a gedankenexperiment about how a tumor can grow and eventually metastasize into its environment from a physics perspective to deduce corresponding mechanical properties. Based on *stiffness, fluidity*, *spatial heterogeneity*, and *texture* of the *tumor front* a roadmap for a prognosis of a tumor's aggressiveness and metastatic potential is presented.

## Graphical abstract

Collective cell motility, a key cancer marker, cannot be measured in vivo. Magnetic resonance elastography can measure its coarse‐grained and emergent impact on tissue mechanics. Combined in vivo and ex vivo analysis of the mechanics of solid tumors identifies increased tumor resistance and tissue fluidity as a pathological signature and a potential prognostic marker of tumor aggressiveness and metastatic potential.

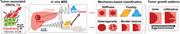

## 1 Introduction

Cancer is a leading cause of death worldwide.^[^ ^1^ ^]^ With an ever‐growing and aging population, a drastic increase in cancer cases is expected within the next 20 years.^[^ ^2^ ^]^ Precise diagnosis and monitoring of a solid tumor are key for individualized treatments to prevent over‐ and undertreatments that can reduce the life expectancy and quality of life of millions of patients worldwide and cause high health‐care costs in the billions.^[^ ^3^ ^]^ Currently, there are more than 150 known cancer types originating from a multitude of tissues,^[^ ^4^ ^]^ and tumors often develop into heterogeneous tissues with many different subpopulations of cells. Therefore, the molecular complexity restricts a comprehensive understanding solely from a molecular perspective. A reductionist, complementary mechanical view that is agnostic to molecular details may help with this problem. Oncology has set out to find the gene signatures of cancer, and even though remarkable progress has been achieved, there is still an urgent need to improve the prognostic and predictive power of expression tests in individual patients further since survival rates have not increased,^[^ ^5^, ^6^, ^7^ ^]^ and less than one‐quarter of cancer patients have benefited from these molecular insights.^[^ ^8^ ^]^ Despite their vast genetic variability, tumors obey the laws of physics by generating forces that displace surrounding tissue or enable cancer cells to spread.^[^ ^9^ ^]^ Currently, cell motility in dense tissues, a prerequisite for metastasis, is not detectable by noninvasive imaging techniques, specific molecular markers or as part of gene signatures. Molecular tumor biology discusses the epithelial‐to‐mesenchymal transition (EMT) as the onset of cancer cells becoming motile in carcinoma.^[^ ^10^, ^11^ ^]^ However, cancer cells do not show a clear, complete transition; instead, they assume a spectrum of transitional states^[^ ^12^, ^13^, ^14^ ^]^ and E‐cadherin is even required for metastasis in multiple breast cancer models.^[^ ^15^ ^]^ Thus, a molecular state defining cell motility has not yet been found. This lack of diagnostic tools enhances the risk of cancer to become a systemic disease that swamps normal tissue and overruns the body with metastatic cells. However, the physical rules of cancer in vivo are still understudied and not yet exploited in oncology.

While the classical mechanical view of a malignant tumor as a stiff, rigid and palpable lump has not significantly changed over the last 3500 years,^[^ ^16^ ^]^ recent studies have also shown cancer cell softening,^[^ ^17^ ^]^ fluidization of cancer cell clusters in tumors^[^ ^18^ ^]^ and malignant brain tumors being softer than surrounding tissue and benign tumors.^[^ ^19^ ^]^ The growing and spreading behavior of tumors is also dependent on their environment. On a single cell level, tumor cells tend to be more mechanically heterogenous with a shift towards softer cell than their healthy counterparts.^[^ ^20^ ^]^ In the cancer cell clusters of primary tumors, softening of cancer cells promotes an unjamming transition, making small areas fluid‐like and enabling cancer cells to change position and migrate.^[^ ^21^ ^]^ In general, cells in the adult body are thought to behave primarily static. They undergo a fluid to solid transition by jamming.^[^ ^22^ ^]^ However, during cancer progression, tumor cells are known to increase proliferation, become mobile and move collectively,^[^ ^23^ ^]^ which cause a shift to a more fluid tissue. On the collective cellular level, the hypothesis that cell movement can be described as cellular unjamming^[^ ^24^ ^]^ has become a key focus in the physics of cancer, suggested by many experimental studies.^[^ ^18^, ^21^, ^25^, ^26^, ^27^ ^]^ From a developmental biology point of view, most tissues can be considered jammed. Nevertheless, they are close to the unjamming transition, which can be triggered by pathological changes.^[^ ^22^ ^]^ This unjamming was found to be driven by critical changes in cell shape and density in cell layers,^[^ ^26^, ^27^ ^]^ multicellular tumor spheroids,^[^ ^21^, ^25^ ^]^ and primary tumor explants.^[^ ^18^, ^21^ ^]^

Unjamming of cancer cells and collective migration appear to be critically important in the malignant transformation of solid tumors as an early event of the metastatic cascade. Cancer cells in a solid tumor can divide against an opposing rigid environment, but only the collective fluid properties enable them to change position and start to move and metastasize.^[^ ^28^, ^29^ ^]^ If cancer‐cell softening induces elevated multicellular motility, the corresponding region will behave more fluid‐like.^[^ ^30^, ^31^ ^]^ Consequently, changes in collective fluid properties of suspicious lesions can provide information on the tumor's metastatic potential and to which extent it has changed during therapy.

This tissue *fluidity* manifests in the dissipation of shear stress energy on a coarser, macroscopic level, which is relevant to current magnetic resonance elastography. In the macroscopic picture, a sample behaves like a solid if it is purely elastic. This means that after removing an external load, the material restores its original shape and returns the stored energy completely. Conversely, a tissue behaves like a fluid if it is purely viscous, meaning that after removing an external load, the material stays deformed, and the external energy is lost due to internal friction or other dissipative processes. Notably, fluid behavior is not necessarily related to the content of water or liquids in a sample. Instead, it is related to the ability of particles such as cells to randomly change position and to dissipate by its motion the deforming stress. Intriguingly, sand becomes sticky or solid‐like when wet and flows fluid‐like through an hourglass when dry. In viscoelastic terms, the solid‐fluid behavior of a material is expressed in its complex shear modulus G*. More specifically, it is expressed in the ratio of its imaginary part, the loss modulus *G*″ to its real part, the storage modulus *G*′. By definition, a tissue is described as solid‐like when the storage modulus exceeds the loss modulus. Vice versa, it becomes more dissipative or fluid‐like when the loss modulus dominates. The phase angle of the complex shear modulus

(1) $$\varphi =\tan^{-1}\left(\frac{G^{\prime \prime }}{G^{\prime }}\right)$$

displays two limits, where *φ* = 0 represents pure solids and *φ* = π/2 represents purely fluid behavior. Hence, an increase of *φ* within this range indicates an increase in the fluid properties of a tissue. A transition from primarily solid to predominantly fluid properties occurs at *φ* *_0_ = π/4*, or in other words; when *G*″ > *G*′. Thus, we define the term

(2) $$\mathrm{fluidity}=\frac{\varphi -\varphi_{0}}{\varphi_{0}}$$

which can range from pure solids at −1 to pure liquids at 1. We define a solid‐fluid transition at 0 (i.e., at *φ*_0_), where values around 0 exhibit characteristics of both solids and fluids. In the future, this range may be more precisely specified, e.g., by ROC analysis of larger data sets.

Magnetic resonance elastography (MRE) can measure the mechanical properties expressed by the dynamical shear modulus G* in soft tissues in vivo, which describes elastic resistance in terms of the storage modulus *G*′ and viscous dissipation in terms of the loss modulus *G*″. *G*′ as measure of elastic strength and the magnitude of the dynamical shear modulus |G*| as a measure of the total mechanical resistance are used in colloquial terms for *stiffness*. It should be noted that MRE cannot distinguish between tumor *stiffness* as an intrinsic material property and extrinsic stress, which affects tumor *stiffness* by increasing elastic stresses and fluid pressure. The latter is subsumed under solid stress and increases tumor *stiffness* in vivo.^[^ ^32^, ^33^ ^]^ In other words, tumors can be equally stiff in vivo and ex vivo, for example, due to high content of fibrous proteins, whereas solid stress due to increased growth pressure in an engulfing tumor niche disappears upon resection, making the tumor softer ex vivo compared to its in vivo state.

Besides *stiffness*, MRE quantifies mechanical dissipation *G*″ and tissue fluidity *φ* as measures of the *fluid behavior*. A MRE setup basically consists of a MRI system that obtains motion fields in tissues using phase‐sensitive sequences, an acoustic actuator hardware that emits harmonic shear waves into the body, and postprocessing to generate mechanical parameter maps.^[^ ^34^ ^]^ Recently, multifrequency MRE has been established as a variant of MRE that provides high‐resolution maps of *stiffness* and *fluidity*. Such a tomographic representation of mechanical tissue properties, known as tomoelastography, opens up the venue for noninvasive, physics‐based cancer diagnosis. In brief, tomoelastography is a recovery mode for maps of viscoelastic parameters in tissue, including *fluidity* and by utilizing wave field inversion at multiple drive frequencies, the resulting parameter maps are less prone to single frequency artifacts and enable their robust and detailed spatial analysis.^[^ ^35^ ^]^ Several studies have already demonstrated the potential that MRE adds diagnostic value to imaging‐based cancer diagnosis. For instance, in the diagnosis of pancreatic ductal adenocarcinoma, the inclusion of MRE‐quantified stiffness alongside contrast‐enhanced CT and MRI significantly improved the overall diagnostic performance, surpassing the use of MRI alone.^[^ ^36^ ^]^ Similarly, in the diagnosis of breast lesions, the combined utilization of MRE and MRI parameters led to improved diagnostic accuracy in comparison to relying solely on the MRI‐based BI‐RADS system.^[^ ^37^ ^]^ The added value of incorporating both *stiffness* and *fluidity* information from tomoelastography has also been demonstrated in the diagnosis and characterization of prostate cancer,^[^ ^38^ ^]^ rectal cancer,^[^ ^39^ ^]^ and hepatocellular carcinoma.^[^ ^40^, ^41^ ^]^

We hypothesize that tomoelastography, measures the mechanical fingerprint of tumors in vivo and is sensitive to microscopic physical interactions, including unjamming of cancer cells. In the following study, we combine vital microscopy of cancer cells and MRE experiments in patient derived tumor explants with tissue‐mimicking collagen samples, along with literature data on in vivo MRE in tumors to explore tissue *stiffness* and *fluidity* as novel potential tumor markers. In the first part of our study, a retrospective meta‐analysis of in vivo MRE studies,^[^ ^19^, ^38^, ^42^, ^43^, ^44^, ^45^ ^]^ we identify general patterns of increased *stiffness* and *fluidity* in tumors with respect to their specific host tissues. We then review mechanisms associated with tumor progression and discuss how they might influence *stiffness* and *fluidity* in affected tissues. Utilizing microscopic analysis and live cell tracking in human tumor explants as well as novel tabletop MRE, we experimentally explore our hypothesis in cellular systems and noncellular microenvironments and focus on emergent effects arising from the collective interplay. Based on these experiments and in vivo MRE results, we perform a gedankenexperiment about the early mechanical prerequisites in primary tumors for cancer progression. We summarize these considerations in a roadmap for noninvasive tumor classification based on multiparametric tomoelastography imaging. Overall, our aim is to translate the principles of cancer mechanics, particularly tissue *stiffness* and *fluidity*, into prognostic imaging markers of tumor aggressiveness and treatment response.

## 2 Experimental Section

### 2.1 In Vivo Tomoelastography and Derived Stiffness and Fluidity Parameters

Tomoelastography exploits mechanical vibrations of frequencies as specified in **Table** **1**. The vibrations were induced by external drivers based on piezoelectric elements or compressed air. 3D wave fields were acquired using single‐shot, spin‐echo echo‐planar imaging sequences comprising flow‐compensated motion‐encoding gradients (MEG). The acquired complex MRI data was smoothed, unwrapped, and a temporal Fourier transformation was applied. The resulting complex‐valued harmonic wave images were further processed for the brain using multifrequency dual elasto‐visco (MDEV) inversion.^[^ ^46^ ^]^ For other body regions, k‐MDEV inversion was used.^[^ ^47^ ^]^ Both processing pipelines are publicly available at https://bioqic‐apps.charite.de. MDEV provides a frequency‐averaged magnitude of the complex shear modulus |G*| by directly solving the Helmholtz equation in magnitude representation.^[^ ^35^ ^]^ Meanwhile, k‐MDEV recovers frequency‐compounded maps of shear‐wave speed *c* as a surrogate parameter of tissue *stiffness*. Only MDEV directly delivers the *fluidity* parameter *φ*; hence both pipelines are combined to recover *stiffness* and *fluidity* in all organs except the brain. In the literature, *stiffness* is discussed based on |G*| or *c*. Both parameters can be converted according to^[^ ^34^ ^]^ into each other via

(3) $$\left|G^{*}\right|=c^{2}\cdot \left[\rho \cdot \left(1+\cos\varphi \right)\right]/2$$

assuming unit density *ρ* = 1 kg l^−1^ giving a good representation of most soft tissues^[^ ^48^ ^]^

**Table 1:** Studies used for meta‐analysis, along with their respective publications and setup parameters. The MDEV pipeline was used for processing brain data, while the k‐MDEV inversion was used for all other organs. Both can be accessed at https://bioqic‐apps.charite.de

Study	Entity/organ	*B*/*T*	Frequency [Hz]	Resolution [mm]^3^
Shahryari et al. 2019^[^ ^42^ ^]^	Liver	1.5	30, 40, 50, 60	3×3×5
Streitberger et al. 2020^[^ ^19^ ^]^	Brain	3.0	30, 35, 40, 45, 50, 55, 60	2×2×2
Zhu et al. 2020^[^ ^43^ ^]^	Pancreas 1	3.0	30, 40, 50, 60	2×2×2
Marticorena et al. 2020^[^ ^44^ ^]^	Pancreas 2	1.5/3.0	30, 40, 50, 60	2.7×2.7×5 / 2×2×2
Asbach et al. 2020^[^ ^45^ ^]^	Prostate 2	3.0	60, 70, 80	2×2×2
Li, Guo et al. 2021^[^ ^38^ ^]^	Prostate 1	3.0	40, 50, 60, 70	2×2×2
Hu, Guo et al. 2021^[^ ^39^ ^]^	Colorectum	3.0	40, 50, 60, 70	3×3×5

### 2.2 Studies Used for Classification

As *tomoelastography* is still an emerging field, there is limited data available on tumors in general (see also ref. [49]), and especially when considering tissue *fluidity*. Therefore, the underlying principles of tumor mechanics were approached agnostically and the data sets were analyzed from all available in vivo tomoelastography studies on solid tumors that included *stiffness* and *fluidity* maps of both tumors and respective control tissue. An overview of the studies and their respective publications can be found in Table 1. A detailed explanation of patient and tumor entity selection is provided in the corresponding studies. The measurements were conducted in clinical 1.5 or 3 Tesla MRI scanners, and all patients underwent both multifrequency MRE and standard clinical T1‐ and T2‐weighted MRI. Regions of interest (ROIs) were taken over the entire tumor entity. The k‐MDEV pipeline was used for data processing in all studies except for Streitberger et al.,^[^ ^19^ ^]^ where the MDEV pipeline was used. Further information about the extracted viscoelastic data and the selection criteria for the control tissues can be found in **Table** **2**. In the brain studies, *stiffness* was originally given as |G*| in.^[^ ^19^ ^]^ For further analysis the shear wave speed *c* was calculated from the corresponding |G*| and *fluidity* according to Equation (3). As this study relied on already published and peer‐reviewed data, the bulk *stiffness* and *fluidity* values of tumor and corresponding reference tissue could only be extracted.

**Table 2:** Mechanical data from previous studies on different tumor entities. HCC: hepatocellular carcinoma, HCA: hepatocellular adenoma, CCA: cholangiocellular carcinoma, FNH: focal nodular hyperplasia HEM: hemangioma, GB: glioblastoma, MEN: meningioma, Ca: carcinoma (for prostate, pancreas, and colon‐rectal entities, DTT: distal tumor‐adjacent tissue, CLH: white matter from the contralateral hemisphere, HV: healthy volunteers, BPH: benign prostatic hyperplasia, PZ: peripheral zone, TZ: transition zone, *converted from |G*| and *φ* to shear wave speed *c* according to Equation (3). °Not from the same patient whose tumor tissue was analyzed

Tumor entity / study	Sample (origin of control tissue)	Stiffness *c* in [m s^−1^]	Fluidity (*φ* − *φ_0_)/φ_0_*	*N*	Study
Liver – HEM (vascular tumor)	Tumor	1.97 ± 0.45	0.21 ± 0.38	11	Shahryari et al.^[^ ^42^ ^]^
	Control (DTT)	1.37 ± 0.13	−0.24 ± 0.04		
Liver – HCA	Tumor	1.41 ± 0.21	−0.16 ± 0.15	15	
	Control (DTT)	1.38 ± 0.12	−0.16 ± 0.08		
Liver – FNH	Tumor	2.08 ± 0.96	−0.07 ± 0.31	10	
	Control (DTT)	1.40 ± 0.12	−0.16 ± 0.05		
Liver – CCA	Tumor	2.57 ± 0.90	0.58 ± 0.32	12	
	Control (DTT)	1.72 ± 0.29	−0.16 ± 0.13		
Liver – HCC	Tumor	2.54 ± 0.64	0.53 ± 0.37	22	
	control (DTT)	1.97 ± 0.49	0.03 ± 0.20		
Brain – MEN	Tumor	1.40 ± 0.32*	0.27 ± 0.17	9	Streitberger et al.^[^ ^19^ ^]^
	Control (CLH)	1.39 ± 0.05*	−0.26 ± 0.09		
Brain – GB	Tumor	1.07 ± 0.28*	−0.54 ± 0.13	9	
	Control (CLH)	1.42 ± 0.18*	−0.17 ± 0.05		
Pancreas – pancreatic cancer / 1	Tumor	2.35 ± 0.39	0.53 ± 0.17	40	Zhu et al.^[^ ^43^ ^]^
	Control (HV°)	1.32 ± 0.05	0.03 ± 0.05	10	
Pancreas – pancreatic cancer / 2	Tumor	2.08 ± 0.38	0.63 ± 0.09	72	Marticorena et al.^[^ ^44^ ^]^
	Control (HV°)	1.28 ± 0.14	−0.01 ± 0.09	30	
Prostate – PCa / 1	Tumor	3.4 ± 0.6	0.66 ± 0.25	73	Li, Guo et al.^[^ ^38^ ^]^
	Control (HV°)	2.1 ± 0.24 (PZ) 2.2 ± 0.1 (TZ)	0.02 ± 0.13 (PZ) 0.14 ± 0.13 (TZ)	53	
Prostate – PCa / 2	Tumor	3.1 ± 0.6	0.40 ± 0.13	14	Asbach et al.^[^ ^45^ ^]^
	Control (BPH°)	2.8 ± 0.4 (PZ) 2.8 ± 0.3 (TZ)	0.15 ± 0.25 (PZ) 0.15 ± 0.13 (TZ)	25	
Colorectum – Colorectal Ca	Tumor	2.3 ± 0.5	0.71 ± 0.36	80	Hu, Guo et al.^[^ ^39^ ^]^
	Control (DTT) Control (HV°)	1.3 ± 0.1 1.4 ± 0.1	−0.087 ± 0.10 −0.06 ± 0.13	80 12	

### 2.3 Tabletop MRE in Collagen Networks

In order to investigate the effects of matrix architecture and crosslinking on the bulk *stiffness* and *fluidity* of an extracellular matrix (ECM) surrogate, 28 samples of collagen type 1 gels were investigated with varying matrix architecture and crosslinking states, but the same concentration, using tabletop MRE. The collagen samples used were either bovine‐skin type 1 (Stock solution: 4 mg mL^−1^, Lot#0235G, Biochrom, Berlin, Germany) or rat‐tail type 1 (Stock solution: 4 mg mL^−1^, Lot# 190594, Serva, Heidelberg, Germany) collagen gel, with a final gel concentration of 3 mg mL^−1^. Details on the collagen gel preparation process can be found in the appendix. The tabletop MRE setup consists of a compact MRI scanner (0.5 T permanent magnet, 10 mm bore) with a system controlled piezoelectric driver placed on top of the sample tube. The glass tubes containing the collagen samples were positioned within the bore of the MRI scanner, which was heated to 37 °C. Vibrations from the piezo actuator were induced via the glass walls into the samples. A more detailed overview of the setup and imaging process can be found in the appendix. A frequency range from 200 Hz to 2 kHz was covered in 100 Hz intervals. The acquired data were unwrapped and Fourier‐transformed in time to extract complex‐valued wave images for each driving frequency. Wave profiles for deflection parallel to the cylinder axis were created and fitted by the analytical solution of shear waves in a *z*‐infinite cylinder,^[^ ^50^ ^]^ resulting in the complex wave number k* = k′ *+ ik*″. Shear wave speed *c* and the shear wave penetration rate *a* were derived from k* for each frequency

(4) $$c=\frac{2\pi f}{k^{\prime }}\;\;\text{and}\;a=\frac{f}{k^{\prime \prime }}$$

A viscoelastic fractional element model based on a generalized fractal Maxwell model,^[^ ^51^ ^]^ that has shown good applicability on both eukaryotic cells^[^ ^52^ ^]^ and biological tissues,^[^ ^51^ ^]^ directly fitted these parameters to derive shear modulus‐related parameters

(5) $$G^{*}=\mu^{1-\alpha }\;\eta^{\alpha }{\left(i2\pi f\right)}^{\alpha }$$

Depending on the power law variable *α*, this model can interpolate between a pure elastic solid with the spring constant *µ* resembling *G*′ for *α* → 0 and a viscous fluid with the dashpot parameter *η* for *α* → 1.^[^ ^51^ ^]^ Since in *µ* and *η* are linearly dependent in Equation (5), we set *η* to 1 Pa s to obtain a 2‐parameter model. The power law variable *α* can be directly translated to the phase angle *φ* of the complex shear modulus G* by multiplying it by π/2, thus representing the *fluidity* of the sample. The *stiffness* can be calculated from the model parameters according to Equations (5) and (3) in terms of the shear wave speed *c* for a given frequency. Here the frequency of 500 Hz was chosen, which is in the middle of the range of 200–800 Hz applied to all samples. Additionally, measurements on 3.0 mg mL^−1^ collagen gels mixed from rat‐tail and bovine‐skin (mixture 1:2) with and without additional glutaraldehyde crosslinking were reevaluated from prior work^[^ ^53^ ^]^ in the above‐described manner and depicted in Figure 4.

### 2.4 Confocal Microscopy of Collagen Networks and Primary Human Tumor Explants

Collagen gels were prepared in plates with thin bottoms suitable for confocal microscopy and fluorescently stained overnight. Image cubes were recorded using a confocal laser scanning microscope (TCS SP8, Leica, Wetzlar, Germany). The analysis of primary human tumor tissue was approved by the ethics committees of the Medical Faculty of Leipzig University (No. 073‐13‐11032013). Tumor tissue was provided by the Institute of Pathology of Leipzig University Hospital immediately after surgery. Pieces from a stage 2 ductal invasive breast carcinoma from a 48‐year‐old woman were analyzed. Histology slices were provided by our collaborators from the pathology department of the Leipzig University Hospital. Detailed information on the sample can be found in the supplementary information. These samples were cut with a scalpel into millimeter‐sized pieces and transferred into plates suitable for confocal microscopy. The tissue pieces were cultured in standard cell culture medium and fluorescently stained for DNA and Actin. 3D timelapse imaging was performed every 10 min over the course of 12 h using a spinning disc confocal microscope (Axio Observer, Zeiss, Jena). Further details can be found in the appendix.

## 3 Results

### 3.1 Tomoelastography Reveals Tumors In Vivo

**Figure 1:**
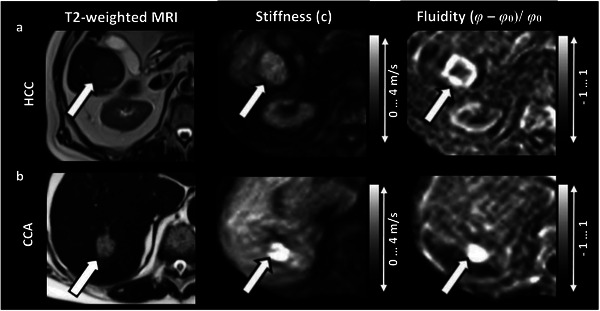
Tomoelastography results for two representative cases of malignant liver tumors. Shown are maps of *stiffness* (shear‐wave speed *c*) and *fluidity* (phase angle (*φ* – *φ*_0_)/*φ*_0_) along with T2‐weighted MR images. a) Hepatocellular carcinoma (HCC, arrow) in liver segment VI in a 78‐year‐old man, revealing high *stiffness* and *fluidity* surrounded by softer tissue. b) 72‐year‐old man with cholangiocarcinoma (CCA, arrow) with larger *stiffness* than nontumorous liver tissue. The *fluidity* in CCA is larger than in nontumorous liver tissue. Image was taken and edited from Shahryari et al. 2019^[^ ^42^ ^]^.

In all seven of the published studies that we have reanalyzed, changes in *stiffness* and *fluidity* were found that characterized tumors relative to the host tissue. This is demonstrated in **Figure** **1** by a side‐by‐side comparison of classical MRI (left), MRE obtained *stiffness* (middle) and *fluidity* (right) for two cases of malignant liver tumors. With respect to *stiffness*, *fluidity* achieves a visibly better contrast in Figure 1. Here the k‐MDEV inversion (see above) was used, which yields the shear wave speed *c* as *stiffness* parameter. Since most of the publications discussed in this study, use this method, we will continue to express *stiffness* in the form of the shear wave speed *c*, although it can be easily converted into |G*| according to Equation (3).

### 3.2 Rheological Fingerprints of Tumors In Vivo

**Figure 2:**
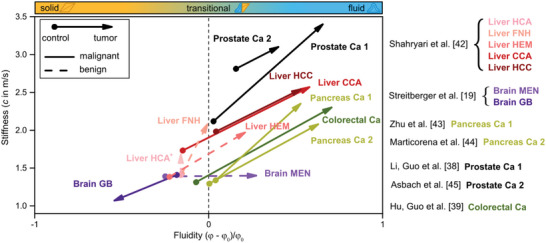
In vivo MRE reveals changes in *fluidity* versus *stiffness* for various tumor entities taken from studies listed above in Table 2. The heat map atop of the figure shows the transition from solid (yellow) to fluid (blue) material properties. The initial point marker of the arrows represents *stiffness* and *fluidity* for the reference tissue, while the arrowhead indicates the mechanical properties of the corresponding tumor. The arrows are color coded to present the published studies given on the right side. All entities, except for the head tumors, show a significant increase in *stiffness* and *fluidity*. The dashed vertical line indicates the transition from solid to fluid material properties. HCC: hepatocellular carcinoma, HCA: hepatocellular adenoma, CCA: cholangiocellular carcinoma, FNH: focal nodular hyperplasia HEM: hemangioma, GB: glioblastoma, MEN: meningioma, Ca: carcinoma (for prostate, pancreas, and colorectal entities), ^+^For Liver HCA, the *stiffness* difference was increased by a factor of 10 for better visibility. The numbers behind the names indicate multiple studies per entity according to Table 2.

For all the tumor entities listed in Table 1, *stiffness* and *fluidity* were extracted and compared to the corresponding reference tissue in Table 2. The reference tissue varied among the studies, ranging from distal tumor‐adjacent tissue (DTT) to unaffected organs from healthy volunteers (HV), as described in Table 2, where in the sample column the corresponding control group is described in brackets. Unless otherwise stated, *stiffness* and *fluidity* of a particular tumor entity were always compared with control tissue from the same study, obtained with the same imaging parameters, etc. With the exception of the benign hepatocellular adenoma (HCA), all the analyzed tumors showed a statistically significant difference in tissue *stiffness* and *fluidity* with respect to their reference tissue, demonstrating altered mechanical behavior in pathologic tumor tissue. Apart from brain tumors, all other tumor entities showed a significant increase in *stiffness* and *fluidity* compared to their reference tissue. Benign liver tumors^[^ ^42^ ^]^ showed less pronounced changes than the malignant entities, with only the benign hemangioma (HEM), a vascular tumor, exceeding the threshold towards a fluid behavior. Also prostate tumors (PCa1)^[^ ^38^ ^]^ showed this significant increase in *stiffness* and *fluidity*, regardless of whether the peripheral zone (PZ) near the rectum or the transition zone (TZ) surrounding the urethra of healthy volunteers was used as a reference. This is further confirmed by a second study on prostate tumors (PCa2),^[^ ^45^ ^]^ where the reference group consisted of patients with benign prostate hyperplasia (BPH), which already leads to early pathologic changes in the tissue and explains the stiffer and more fluid reference tissue compared to younger and healthy controls (HV) in.^[^ ^38^ ^]^ Cases of benign prostate hyperplasia (BPH) were also included in the first mentioned publication on prostate tumors (PCa1)^[^ ^38^ ^]^ and showed with (2.6 ± 0.3) m s^−1^ and 0.27 ± 0.25 similar tissue *stiffness* and *fluidity* in relation to the reference values from PCa2.^[^ ^45^ ^]^ The transition zone (TZ) surrounding the urethra is more susceptible to BPH, while prostate cancer occurs mainly in the peripheral zone (PZ) near the rectum. The study on pancreas tumors (Pancreas Ca 1)^[^ ^43^ ^]^ shows the same trend, confirmed by a second study (Pancreas Ca 2)^[^ ^44^ ^]^ on the same tumor entity. The study on colorectal cancer^[^ ^39^ ^]^ also ratified the observed trend. Conversely, brain tumors^[^ ^19^ ^]^ displays an inverted mechanical behavior. While the benign brain tumor, meningioma (MEN), predominately showed an increase in *fluidity* compared to healthy brain tissue from the contralateral hemisphere (CLH), the malignant glioblastoma (GB) showed both a significant decrease in *stiffness* and, most notably, a drastic drop in *fluidity* in comparison to the CLH. The results of this meta‐analysis are summarized in **Figure** **2**, and the relative differences in *stiffness* and *fluidity* between tumor and corresponding control tissue for the individual studies are depicted in Figure S1 (Supporting Information).

### 3.3 Partial Tissue Fluidization in Cancer Cell Clusters in Primary Human Tumor Explants Causes a Spatially Heterogeneous Fluidity

**Figure 3:**
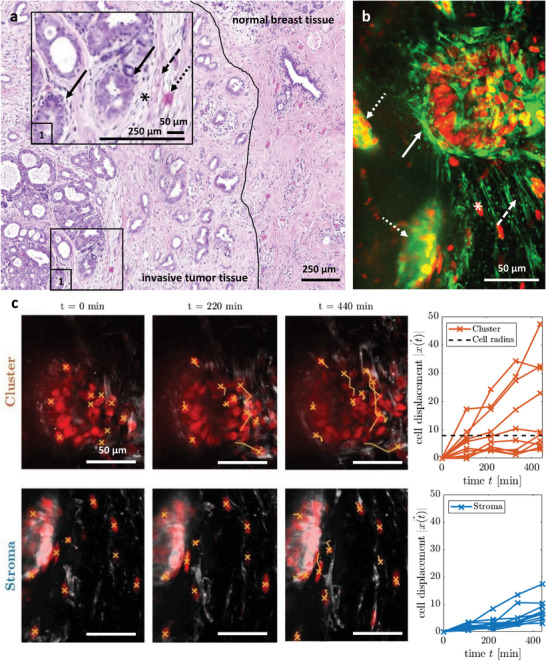
Fluid‐like and solid‐like behavior in a primary human breast carcinoma. a) representative H&E‐stained histological slice showing invasive tumor tissue on the left and normal breast tissue on the right. The insert (1) gives a more detailed view on an area with high cell density, identifying cell clusters (black solid arrows), surrounding stromal tissue (dashed black arrows) with single cells (*), presumably cancer associated fibroblasts (CAFs). The brighter pink color further indicates small blood vessel (dotted black arrow). b) Snapshot (maximum intensity projection) of vital tissue from a different tissue explant from the same tumor shows a cluster of cancer cells (bold white arrow) that is connected via elongated cells (asterisks) to a low‐cellular, most likely stromal area (dashed arrows) indicated by fiber‐like orientation of the cells green actin signal. Several vessel‐like structures are visible in the background of the image volume (white dotted arrow). Manually dissected pieces of the tumor were stained with a vital DNA stain (red SPY650‐DNA) and a vital F‐actin stain (green, SPY555‐Actin). Z‐projection of 180 µm (20 image slices) recorded after an experimental runtime of 36 h. c) Vital nucleus tracking showed cells with high motility in the cluster‐like region (top row) in contrast to slower single cells in the stroma‐like region (bottom row). Tracking of 10 representative cells was done manually in Matlab. Scale bars: 50 µm. The cell displacements for these tracks are plotted on the right for each region. For the cluster region we additionally plotted the typical cell radius as a threshold for fluid‐like behavior. This criterion does not apply to single cells as tracked in the stroma like region.

Cells in densely packed cell clusters can collectively transition from solid‐like to fluid‐like behavior, a phenomenon known as unjamming.^[^ ^54^, ^55^ ^]^ This process is associated with tissue fluidization and is believed to be crucial in tumor progression, increasing the metastatic risk; and other diseases.^[^ ^21^, ^26^, ^30^, ^56^ ^]^ However, tumors are not composed only of cell clusters; they are complex compound materials, where the cancer cell clusters are typically surrounded by fibrotic extracellular matrix. To assess the micro‐fluid behavior of tumors defined by particles (e.g., cancer cells) that can change position, we present an intact explant of a primary human breast tumor that in contains in a cell cluster both unjammed motile cell regions and jammed, solid ones with nonmotile cells. **Figure** **3**a depicts a H&E‐stained histological slice taken after surgical removal of the tumor showing tumor tissue at the left and normal breast tissue on the right. The black line marking the border was drawn by a senior pathologist. The insert shows a magnification of an area with a high cellular density marked with (1). Black arrows indicate cell clusters, while the dashed black arrow highlights a stromal area around the tumor where also single cells (*) are found. The dotted black arrow indicates a small blood vessel. An additional H&E slice from the same tumor and their pathological assessment is found in the appendix. We cut millimeter‐sized volumetric vital tissue explants from the same tumor (see Figure S3 in the Supporting Information) for 3D live cell tracking. Figure 3b shows a snapshot (maximum intensity projection) of an area where a dense cell cluster (white arrow) is situated in a stroma‐like area (dashed white arrow) with single cells present (*). Volumetric vessel structures can be seen in the background (dotted white arrows). This snapshot was taken from a 7‐h live observation experiment, which can be seen as Video S1 (Supporting Information). Videos [Link], [Link], [Link] (Supporting Information) show different regions and scenarios from the same tumor sample. Live cell tracking in this dense cell cluster reveals a high activity and mobility (upper row Figure 3c). As displayed by the cell displacements on the far right of Figure 3c, a substantial fraction of tracked cells in this region migrate several cell radii within a few hours and are therefore above the critical threshold of one cell radius typical for caged diffusion within cell clusters,^[^ ^21^ ^]^ characteristic for regions of fluid, unjammed cell aggregates. In our previously published reports, we also found regions of jammed cells in the cancer cell clusters that only perform caged motion.^[^ ^18^, ^21^ ^]^ In contrast to the unjammed cells that we found in cell clusters, in the stromal‐like region next to the cluster (lower row Figure 3b) most of the cells remain quasi stationary, while some are able to slowly migrate through the ECM. For the stroma‐like area we are tracking single cells and therefore the threshold of one cell radius does not apply. The cells in the stroma are inherently motile since they must have moved into the extracellular matrix. Somewhat surprisingly, the unjammed cells move faster than the cells in the stroma, which microscopically indicates a collective, highly fluid behavior. We have recently shown that these fluid regions maintain their mechanical resistance by tension percolation.^[^ ^17^ ^]^ Our findings shown in Figure 3 are consistent with other observations made by our group in this tumor (see Videos [Link], [Link], [Link] in the Supporting Information) and agree with our previous publications.^[^ ^17^, ^18^, ^21^ ^]^ Also in agreement with our recent publications,^[^ ^17^, ^18^, ^21^ ^]^ our results show, that a solid tumor is mechanically *heterogeneous* and comprises both active, fluid‐like regions as well as more static, solid‐like regions.

### 3.4 Tabletop MRE Reveals the Effects of Matrix Crosslinking and Structure on Bulk Stiffness and Fluidity of Collagen Gels

**Figure 4:**
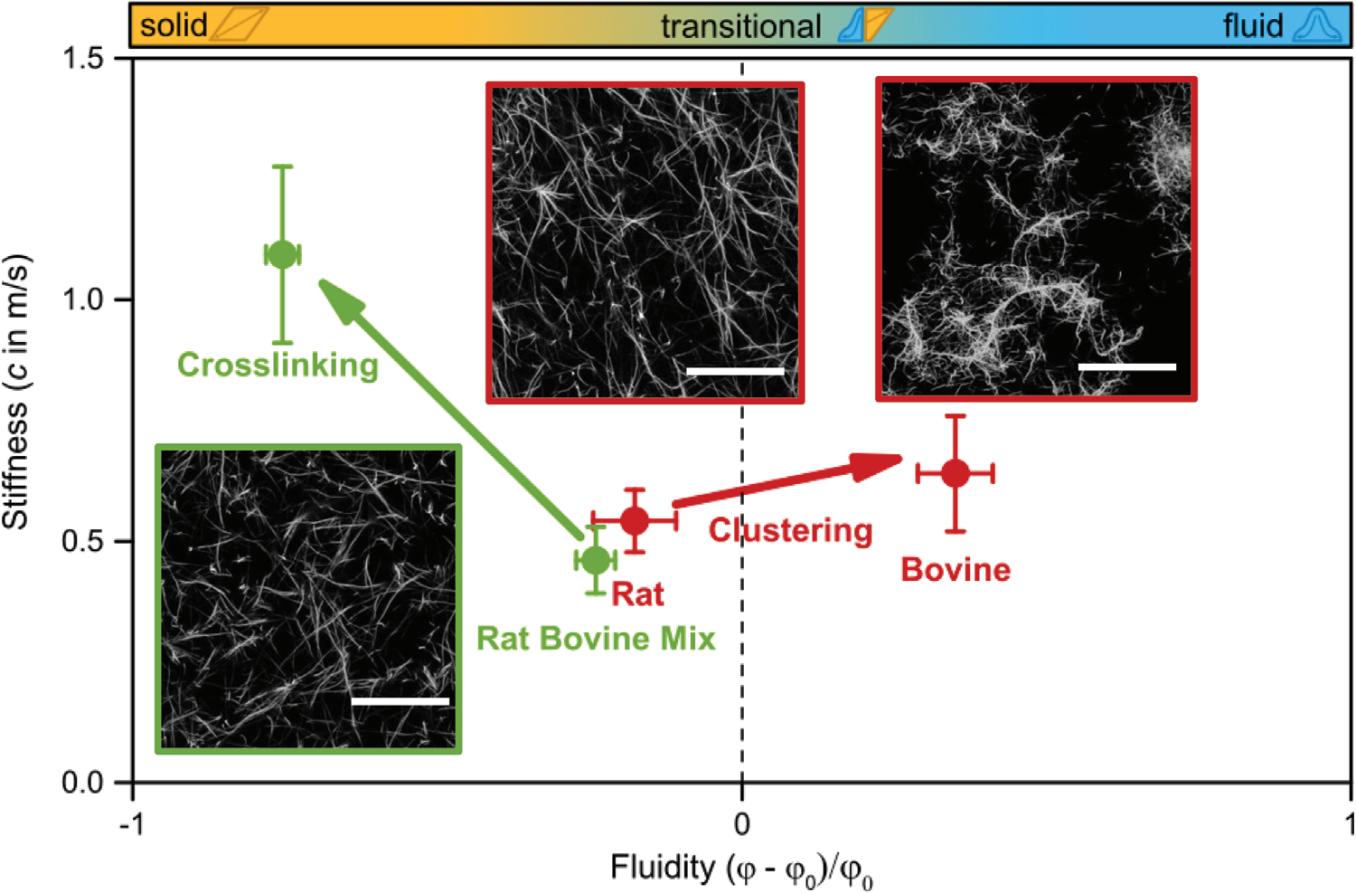
*Stiffness* and *fluidity* of collagen gels of different compositions measured by tabletop MRE. The heat map atop of the figure shows the transition from solid (yellow) to fluid (blue) material properties. Tabletop MRE identified increased fluidity from rat to bovine collagen gels (red), which was associated with a more segregated matrix architecture into clusters confirmed by confocal laser scanning microscopy (CLSM). Rat and bovine type 1 collagen gels were both at 3.0 g L^−1^ concentration. On the other hand, artificial matrix crosslinking does not alter the pore size of the network but reduced fluidity and increases stiffness (green) in 3.0 g L^−1^ collagen gels mixed from 1:2 rat and bovine type 1 collagens Scale bars: 50 µm.

Since a solid tumor is predominantly a compound material of cancer clusters surrounded by fibrotic extracellular matrix, we aim to broaden the understanding of the mechanical behavior of tissues beyond cellular components and examine how the ECM can influence *stiffness* and *fluidity*. To achieve this, we conducted in vitro experiments using pure collagen gels and a tabletop MRE, analyzing them similarly to the in vivo studies presented in Figure 2 above. For each gel structure we had a sample size of *N* = 7 and the collagen concentration was kept at 3.0 g L^−1^. Collagen crosslinking strengthens protein‐protein interactions, increasing s*tiffness* and reducing *fluidity* in the samples. The addition of 0.2% glutaraldehyde solution to a 1:2 mix of rat tail and bovine skin collagen increased *stiffness* from (0.461 ± 0.069) m/s to (1.09 ± 0.18) m/s while reducing *fluidity* from −0.240 ± 0.041 to −0.754 ± 0.034, as shown by the green arrow in **Figure** **4**. We converted these measurement points from our recent study^[^ ^53^ ^]^ with a tabletop MRE, where we also demonstrated that this chemical crosslinking does not affect pore diameter or water diffusion in the matrix (as also seen in the corresponding LSM image in Figure 4 for comparison). This demonstrates that tissue *fluidity* can drastically change without alterations of the hydrodynamic properties of the solvent.

By measuring pure rat‐tail or bovine‐skin collagen gels, we aimed to illustrate the effect of matrix architecture on viscoelastic bulk properties. The *stiffness* of the rat‐tail collagen gel (0.542 ± 0.065) m s^‐1^ was lower than that of the bovine‐skin gels (0.64 ± 0.12) m s^−1^. However, most notably, while the rat tail collagen gels exhibited predominantly solid properties with a *fluidity* of −0.176 ± 0.087, the bovine‐skin gels showed predominantly fluid properties with a *fluidity* of 0.350 ± 0.077. This effect is shown by the red measurement points in Figure 4. Corresponding LSM images (see Figure 4), reveal that rat‐tail collagen forms a more regular and homogeneous network, whereas bovine‐skin collagen gels are characterized by a more heterogeneous network architecture, including vast open spaces and knots (i.e., clusters) with high collagen density. A more detailed analysis of substructural differences can be found in.^[^ ^57^ ^]^ All things considered, our findings demonstrate that even pure collagen networks can exhibit drastically different *fluidity* behaviors, showing a wide range from solid‐like to fluid‐like material properties – even at a constant concentration.

### 3.5 Gedankenexperiment: Under Which Conditions can a Tumor Grow and Eventually Metastasize?

We discovered intriguingly robust trends in changes in the bulk *stiffness* and *fluidity* of solid tumors in contrast to their surroundings. We have also identified potential mechanisms, including ECM alterations and increased motility in dense unjammed cancer cell clusters. Given that these are archetypical tumor features that are also listed in the “Hallmarks of Cancer”^[^ ^23^ ^]^ we are now asking whether the resulting mechanical changes are merely side effects or whether they play an essential role in tumor progression. Are they general prerequisites for the growth or progression of solid tumors? We are aiming towards a mechanics‐based tumor marker system with prognostic potential using the significance of mechanical pathologic changes for metastasis. Although we assume that this approach must also apply to metastatic lesions, we are focusing here on primary tumors that are already the standard target by clinical imaging and thus can be readily integrated into the current clinical workflow. As a reference for pathological changes in tumors that drive cancer progression, we choose the surrounding tissue. This does not only include the direct cellular linage from which the cancer cells stem from. We consider mechanical changes that favor cancer progression with respect to the surrounding tissue. This includes connective tissues and different cellular tissues. If the surrounding tissue is mechanically inaccessible or too small, a surrogate reference tissue, such as the contralateral hemisphere in the brain, can replace it. Further examples are listed in Table 2. Naturally, tumors do not necessarily grow in healthy tissues; the surrounding tissue can already be highly pathologically altered. For example, hepatocellular carcinoma (HCC) almost always have a cirrhotic surrounding host tissue,^[^ ^58^ ^]^ which has an inherently increased *stiffness* and *fluidity* by itself. On a coarse‐grained tissue level, we try to conceive the mechanical differences between a pathological lesion that cannot advance, a benign tumor that can only grow locally, and a malignant solid tumor that invades surrounding tissue and sends out cancer cells.

### 3.6 Tumor Growth as Heterogeneous Tissue Fluidization

In order for a tumor to grow cancer cells must be able to move and proliferate. Cell movements, in particular collective cell motility, inherently contribute to fluid‐like behavior and are essential to dissolving solid‐like structures.^[^ ^21^, ^59^ ^]^ Proliferation also has the potential to fluidize tissues (e.g., by inducing cell unjamming).^[^ ^60^ ^]^ Both proliferation and motility are hallmarks of cancer and are signatures of tumor aggressiveness on different timescales.^[^ ^61^, ^62^ ^]^ Thus, although tumors are often described as *stiff mass* with higher mechanical resistance, central mechanisms also fluidize. This must not be a contradiction since also viscous behavior provides mechanical resistance. Cancer cell motility and proliferation are highly hindered in a solid microenvironment. When we think of tumor progression as the onset of an effective melting process as a requisite for tumor activity, we should expect substantial spatial *heterogeneity* manifested as fluid islands within a still solid background.^[^ ^63^ ^]^ We and others have also shown fluid‐like regions next to solid‐like regions in tumor tissues, further corroborating the concept^[^ ^17^, ^21^ ^]^ that a tumor has a solid‐like backbone and interspersed fluid areas in which motile cancer cells display liquid‐like stream patterns.^[^ ^64^ ^]^ Moreover, tension percolation in the fluid regions contributes to the tumor's mechanical resistance. Cancerous tissue can be identified on histological slices from breast tumors by dense cancer cell clusters embedded in stroma‐rich areas.^[^ ^18^ ^]^ Cancer is a dynamic disease, and the cell population in the bulk tumor becomes more heterogeneous over time by newly emerging cancer cell phenotypes.^[^ ^65^ ^]^ Solid tumors also consist of a plethora of other cell types, like granulocytes, macrophages, mast cells, fibroblast, and epithelial cells.^[^ ^66^ ^]^ For example, in glioma, tumor‐associated macrophages can make up to one‐third of the total tumor mass,^[^ ^67^ ^]^ while in other solid tumors, this number can reach up to 50%.^[^ ^68^ ^]^ Naturally, this increased diversity results further in a heterogenization of mechanical parameters. Therefore, we further introduce *heterogeneity* of the spatial *fluidity* distribution as a diagnostic parameter. We expect that in tumor tissue, *fluidity* shows greater spatial variety between fluid, transitional, or solid states than in nonaffected tissue or benign lesions, as well as fluid‐filled cavities such as cysts.

### 3.7 Spreading Types

Some tumors grow rapid and excessively disseminate cancer cells, while others progress slowly or even stay dormant for years.^[^ ^28^, ^69^ ^]^ In our gedankenexperiment, we assume that the surrounding tissue remains static while the tumor tissue can grow and contain motile cells. We make obvious that a tumor's ability to grow in its surrounding environment depends on the relationship between its *stiffness* and *fluidity*. We distinguish between two distinctive tumor growth behaviors. The first behavior we call *displacing growth*, which leads to displacement and potential deformation of surrounding tissue without cellular infiltration. This requires force generation (e.g., through proliferation), creating compressive loads in the tumor and the directly adjacent tissue that allow the tumor to expand in its environment. If these are too strong, nonlinear tumor deformations may lead to additional strain stiffening. *Displacing growth* requires increased tumor tissue *stiffness* to push its surrounding out of the way. This type of growth is possible if the *stiffness* in the tumor generated by elasticity and viscosity outmatches the *stiffness* of the surrounding tissue.^[^ ^60^ ^]^ Conversely, the tumor's ability to invasively expand requires activity (motility, proliferation) that relies on fluidization and on the yielding ability of the surrounding tissue, hence its viscosity or in other words, its *fluidity*. More viscous surrounding brain tissue cannot resist the more solid stress of a growing tumor mass, requiring much less an increased *stiffness* of the tumor in the brain.^[^ ^70^ ^]^ Nevertheless, tumor expansion in the case of *displacing growth* requires an increased tumor *stiffness*. *Displacing growth* describes large bulk deformations of surrounding tissues and is also compatible with benign phenotypes that grow but remain confined.

However, *displacing growth* provides little information about whether cancer cells can escape from the tumor mass as an important early process in the metastatic cascade. Metastasis is more closely linked to processes at the cellular level and less connected to pathologic bulk *stiffness* changes. Cancer cell escape requires active motile cells and regions, which as we showed above (Figure 3a) and elsewhere,^[^ ^21^ ^]^ are connected to tissue fluidization. Therefore, we introduce *infiltrative cell spreading* as the second tumor growth type, characterized by unjammed cancer cells that can migrate within and leave the tumor, e.g., through the surrounding connective tissue or invasion into the lymph or vascular system. At the tumor front this would not manifest by the displacement of the tumor boundary but rather by the dissolution of a clear boundary. *Infiltrative cell spreading* is not linked to the large compressive bulk deformations seen in *displacing growth*, and thus we expect a less central role of bulk *stiffness*. The active, motile cells involved should instead lead to a high *fluidity*. The diffuse boundaries are a typical mechanical signature of this type of growth. A tumor's *infiltrative cell spreading* can sometimes be deduced from its bulk mechanical properties. An example can be found in glioblastoma. The tumor has both a lower *fluidity* and a lower *stiffness* as the surrounding tissue causing a viscous fingering behavior due to the Saffman–Taylor instability, which according to^[^ ^19^ ^]^ predicts unstable borders when

(6) $$\frac{\sin\varphi_{\mathrm{tumor}}}{\sin\varphi_{\mathrm{control}}}<\frac{\left|G_{\mathrm{control}}^{*}\right|}{\left|G_{\mathrm{tumor}}^{*}\right|}$$

However, in many cases a comparison of the bulk properties between tumor and surrounding does not suffice to predict infiltrative cell spreading and a closer analysis of the *tumor front texture* is necessary.

### 3.8 Tumor Front Texture

In passive fluids, a stable smooth border is generated by sufficient surface tension. In cell clusters, there are different mechanisms at work that can create an effective tissue surface tension, which hold back cells from leaving the cluster.^[^ ^56^, ^71^, ^72^ ^]^ If cancer cells can move within the tumor but cannot leave the tumor, a *fluidity* map will reveal a sharp, defined, and clearly delimited border. This *tumor front texture* is linked to *displacing growth*. On the other hand, if cells can leave the tumor in cell strands/aggregates or as individual single cells, an unstable boundary manifests itself as more diffuse, less smooth border with eventual viscous finger‐like structures. We believe that such patterns are archetypical for *infiltrative cell spreading*. It can result from highly active, fluid‐like cells as shown in the measurement in Figure 3a, where cells leave a cancer cell cluster and infiltrate their surroundings. Therefore, diffuse boundaries are also associated with spatial heterogeneity, as explained above, and particularly connected to *fluidity*. Moreover, we think that the tumor front texture itself is indicative of aggressive mechanics. The types of *tumor front textures* we describe are also found in histopathology as distinct growth patterns.^[^ ^73^ ^]^ In histopathological image analysis of cervix carcinoma, these invasion patterns^[^ ^74^ ^]^ and the connected border shape parameters^[^ ^75^ ^]^ have prognostic value. Furthermore, a spray‐like infiltration pattern is positively associated with the presence of more peritumoral desmoplasia^[^ ^76^ ^]^ – the accumulation of fibrous tissue around the tumor.

### 3.9 Roadmap to a Novel Prognostic Tumor Marker

**Figure 5:**
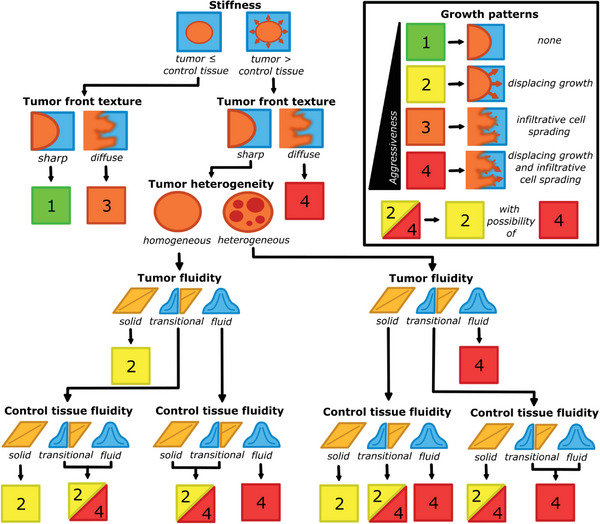
Classification scheme toward prognostic tumor imaging based on tomoelastography. The scheme is derived from our gedankenexperiment and provides a decision tree as flow chart for prognostic tumor imaging based on the 5 input parameters (*stiffness* ratio between tumor and control tissue, tumor *fluidity*, control tissue *fluidity*, tumor *heterogeneity* and *tumor front texture*). The insert connects the spreading types morphodynamically with the resulting growth patters according to their malignancy. Tissues with transitional *fluidity* are considered in transition between fluid and solid material properties and can therefore exhibit both traits, resulting in type 2/4 growth pattern. Figure S4 (Supporting Information) shows a case‐wise classification along increasing imaging resolution.

Based on the gedankenexperiment, we propose a roadmap towards a biomechanical classification scheme for tumor growth based on tomoelastography which results in a growth pattern that takes into account the two spreading types (*displacing growth* and *infiltrative cell spreading*) discussed above and assumes that they can be present independently of one another. These growth patterns are not directly related to patient survival but rather reflect mechanisms related to tumor aggressiveness. Therefore, the four possible growth patterns:

– Type 1 (green): neither *displacing growth* nor *infiltrative cell spreading*
– Type 2 (yellow): only *displacing growth*, no *infiltrative spreading*
– Type 3 (orange): only *infiltrative cell spreading*, but no *displacing growth*
– Type 4 (red): both *displacing growth* and *infiltrative cell spreading*

range from a mainly benign to a highly invasive, aggressive tumor. These are further illustrated in the insert in **Figure** **5**, where also the same color coding as listed above is used. The 4 growth patterns are deduced from the following input parameters: bulk *stiffness* and *fluidity* from both the tumor and surrounding or control tissue, as well as the *heterogeneity* in the spatial *fluidity* distribution of the tumor and the *tumor front texture* between the tumor and surrounding tissue. The two *stiffness* parameters (i.e., bulk *stiffness* of the tumor and bulk *stiffness* of the surrounding tissue) can be condensed into the relation of tumor *stiffness* over surrounding tissue *stiffness*, i.e., whether the tumor is stiffer than the surrounding tissue. The *fluidity* of both tumor and surrounding tissue can be categorized as either *solid*, *transitional*, or *fluid*. The tumor tissue can be either *heterogeneous* or *homogenous* regarding its spatial *fluidity* distribution. In addition, the *tumor front texture* between the tumor and surrounding tissue can be either *sharp* or *diffuse*. These five input parameters result in a total of 72 possible combinations, which are listed in the appendix in detail. Some combinations are straightforward. If the tumor is softer than the surrounding tissue, there is no displacing growth present, and the growth pattern is either type 1 or 3, depending solely on *tumor front texture*. If the *tumor front texture* is diffuse, there is always potential for infiltrative growth, leading to types 3 or 4. When the spatial fluidity distribution of the tumor is labeled as *heterogeneous*, it is automatically associated with at least partial fluid‐like properties – even if the bulk *fluidity* is in the *solid* or *transitional* regime. Other cases are more complex. A graphical overview of our tumor assessment as a function of the input parameters is shown as a diagnostic flow chart in Figure 5. A case‐wise classification along increasing imaging resolution is shown in Figure S4 (Supporting Information).

### 3.10 Retrospective Testing of the Classification Scheme Using the Available Studies

Consequently, we applied this classification scheme to the tumor data presented in Table 1 and Figure 2. The available in vivo MRE data only allowed for the assessment of bulk *stiffness* and *fluidity*, and did not provide the necessary information to evaluate spatial *heterogeneity* of *fluidity* (see also Figure 3a) or the *tumor front texture*. As these parameters are necessary for translating our classification approach into a reliable in vivo tumor marker, we adopted a putative approach by substituting the missing parameters based on histopathological data, our own experiments (as illustrated in Figure 3a) and the gedankenexperiment discussed above. Nevertheless, with the current advancements in multifrequency MRE and tomoelastography, we can conceptually anticipate being able to evaluate most of the parameters in the near future by MRE and tomoelastography. Further details and a critical view on resolution with respect to the *tumor front texture* can be found in the discussion.

The results of our analysis are summarized in **Table** **3** and demonstrate that the classification scheme works well for most cases. For example, colorectal carcinoma is a *fluid* tumor growing in a *solid* to *transitional* environment. Due to its higher *stiffness* than its surroundings, it has the potential for *displacing growth*. Furthermore, its high *fluidity* and diffuse *tumor front texture* enable the second growth type, i.e., *infiltrative cell spreading*, resulting in growth pattern type 4. There are some exceptions, such as the HEM, where due to the high bulk *fluidity* of this benign vascular tumor the resulting growth pattern is type 4 when it should be type 1 or 2. Therefore, type 2 is noted in brackets. In a clinical context, MRE examinations are usually accompanied by classical MRI, where HEMs are easily detected due to their strong T2‐hyperintensity.^[^ ^77^ ^]^ The brain entities MEN and GB are labeled correctly according to their malignancy, i.e., MEN as benign (type 1) and as GB malignant (type 3). MEN are also clinically considered benign tumors. A begin cell mass has been also generated by cell proliferation. Thus, we find slow *displacing growth*, which is slower than in a more aggressive growing tumor (i.e., type 2). With regards to GB, *stiffness* and *fluidity* permit *infiltrative cell spreading*, including viscous fingering (i.e., type 3). Beyond this, GB highly disseminates cancer cells, but can also displace surrounding brain tissue (i.e., type 4), although passively through GB‐induced brain oedema.

**Table 3:** Potential application of the tumor classification scheme from Figure 5 to data from Table 1. Current clinical in vivo MRE only provide bulk *stiffness* and *fluidity* and does not resolve the sample *heterogeneity* and *tumor front texture*. To compensate for this, we added this information putatively according to the explanation in the gedankenexperiment and histopathological findings. Unclear growth patterns are shown with corrections in brackets and are further explained in the text. Redundant (red.), transitional (trans.)

Currently accessible with in vivo MRE				No in vivo MRE data			
Entity	Stiffness tumor/control	Fluidity tumor	Fluidity control	Heterogeneity tumor	Tumor front texture	Growth pattern	Clinical diagnosis
Brain – Necrosis	≤ 1	Fluid	Red.	+ (red.)	Diffuse	3	Malignant
Liver – FNH	> 1	Trans.	Solid	+ (red.)	Sharp	2	Benign
Liver – HCA	≤ 1	Solid	Solid	+ (red).	Sharp	1	Benign
Liver – HEM	> 1	Fluid	Solid	+	Sharp	4 (2)	Benign
Liver – CCA	> 1	Fluid	Solid	+	Sharp	4	Malignant
Liver – HCC	> 1	Fluid	Trans.	+	Sharp	4	Malignant
Brain – MEN	≤ 1	Fluid	Solid/trans.	+	Sharp	1 (2)	Benign
Brain – GM	≤ 1	Solid	Solid/trans.	+	Diffuse	3 (4)	Malignant
Pancreas Ca	> 1	Fluid	Trans.	+	Sharp	4	Malignant
Prostate Ca	> 1	Fluid	Trans./fluid	+	Red.	4	Malignant
Colorectal Ca	> 1	Fluid	Solid/trans.	+ (red.)	Diffuse	4	Malignant

## 4 Discussion

The pathologic changes of tissue in solid tumors exhibit clear trends (see Figure 2) toward *stiffening* and *fluidization*. We argue that these are functional prerequisites to assure bulk tissue growth in tumors as we outlined in our gedankenexperiment (see Figure 5) and thus are essential in tumor progression. But, if tissue fluidization is a biophysical prerequisite for cancer cell motility, why do brain tumors deviate substantially from this narrative? To answer this, we aim to establish a connection between key processes of tumor progression and required, pathologic, mechanical changes in bulk *stiffness* and *fluidity* that can be measured with MRE. We focus on tissue fluidization through unjamming of cancer cells, which causes a transition to cell motility and is an early prerequisite for the metastatic cascade and we could recently demonstrate that unjamming can serve in breast cancer as a tumor marker for metastatic risk.^[^ ^18^ ^]^ Furthermore, it is necessary to investigate and discuss the emergent behavior of cellular unjamming in specific microenvironments to adequately understand the robust trends we see in vivo and the apparent deviations from it. In the following, we discuss these key processes of tumor progression that also include some of the “hallmarks of cancer”,^[^ ^23^ ^]^ which are currently not detectable by medical imaging^[^ ^78^ ^]^ and connect them to tumor biomechanics that we can assess with in vivo MRE.

### 4.1 Tumor Vascularization

With respect to normal vessels in healthy tissue, angiogenesis, and subsequent (neo)vascularization in tumors is described as abnormal, with a high order of disorganization, varying diameters and excessive branching.^[^ ^79^ ^]^ The tendencies to form shunts,^[^ ^80^ ^]^ and defects in their endothelial lining make them leaky.^[^ ^81^ ^]^ Additionally, tumors can form their own cancer cell‐lined vessel, known as vascular mimicry.^[^ ^82^ ^]^ All these factors contribute to fluid accumulation within a tissue, leading to an increase in its viscous dissipation and a more fluid tissue behavior. Thus, consistent with the trend of an increased *fluidity* that we have observed, *fluidity* measurements do not only indicate cancer cell unjamming it also can be the signature of tumor vascularization. Both events are factors for an increasing tumor aggressiveness, which causes pathologic *fluidity* changes in MRE measurements. In some tumor entities, such as the liver hemangiomas (HEM)^[^ ^42^ ^]^ the high tissue *fluidity*, which we see in our analysis (see Figure 2) above, is not due to malignant transformation and is expected to be caused by the already existing high level of vascularization. For other entities prognostic outcome and metastasis can be related to angiogenesis^[^ ^83^ ^]^ and a consequent increase in tissue *fluidity*. Furthermore, the increased interstitial pressure of tumors is also attributed to this liquid accumulation,^[^ ^84^, ^85^ ^]^ contributing to an increased bulk *stiffness*.

### 4.2 Necrosis

Due to insufficient nutrient supply, malignant tumors undergo necrotic cell death, primarily in their core area.^[^ ^86^ ^]^ This does not only increase the amount of free or unbound water in the tumor tissue, but also its viscous dissipation due to the loss of overall structural integrity. Besides the impact on *fluidity*, this also increases the *spatial fluidity heterogeneity* of the tumor tissue. In parameter maps obtained by MRE, necrotic areas, e.g., in the brain (see Figure S5 in the Supporting Information), can be recognized by their typical *heterogeneous* high *fluidity* and simultaneously low *stiffness*. In close agreement, a general reduction of both elasticity (*G*′) and viscosity (*G*″) have been reported for tumor necrosis in animal models.^[^ ^87^ ^]^ Since necrosis only occurs in larger, more advanced tumors, the increase in *fluidity* and *heterogeneity* is again a signature of tumor progression.

### 4.3 Proliferation

While cells in mature tissue usually remain dormant, cancer cells can evade control mechanisms and proliferate unregulated.^[^ ^23^ ^]^ This leads to an increase in cell density^[^ ^88^ ^]^ and over the timespan of month to years, the growth of a primary tumor.^[^ ^89^ ^]^ Proliferation, as well as apoptosis introduce anisotropic stress sources in a tissue that create cellular rearrangement and motility.^[^ ^60^ ^]^ Most importantly, highly proliferative regions of cell aggregates become unjammed and fluid.^[^ ^90^ ^]^ This can effectively transform a tissue from a solid to a viscoelastic fluid,^[^ ^30^, ^31^, ^91^, ^92^ ^]^ increasing its *fluidity*. Over the timescale of weeks to months, uncontrolled cell division and the resulting *displacing growth* requires *fluidity* and the deforming mechanical resistance of the tumor leads to mechanical resistance and thus increasing *stiffness*. Otherwise, the tumor cannot grow under the interplay of apoptosis and proliferation.^[^ ^60^ ^]^ High proliferation is definitely a sign of an aggressive tumor and leads to increased *fluidity*.

### 4.4 Cellular Unjamming

Proliferation induced unjamming should lead to a fluidization on very long time scales, while cell motility transitions based on unjamming should occur much faster. The effect of cell motility‐induced cell unjamming takes place within a much shorter timescale, typically ranging from minutes to hours. Unjamming has been demonstrated to cause tissue fluidization in various scenarios, including cell culture,^[^ ^93^ ^]^ embryogenesis,^[^ ^59^ ^]^ asthma,^[^ ^26^ ^]^ and cancer.^[^ ^18^, ^21^ ^]^ In the case of cancer, we have found highly motile fluid‐like regions in cancer cell clusters of explants from primary human tumors, as depicted in Figure 3a and other previous references cited, all showing an increase in *fluidity*. In the Self‐Propelled Voronoi (SPV) model, this fluidization is realized by a vanishing shear modulus,^[^ ^24^ ^]^ leading to a decrease in *stiffness* by showing no elastic resistance. In contrast, recent experimental evidence points towards tissue fluidization by increased traction and propulsion, allowing cells to squeeze through tissues.^[^ ^94^, ^95^ ^]^ Tension percolation leads to a finite shear modulus and some elastic behavior.^[^ ^17^ ^]^ Unlike unjamming in the SPV model, this mechanism raises mechanical resistance of the tumor tissue, as opposed to relaxing residual stresses.^[^ ^96^ ^]^ This may result in a fluid tissue that is rather stiff than soft. However, the specific mechanical resistance depends on the ratio of unjammed to jammed cells in the cancer cell clusters. According to our previous study,^[^ ^17^ ^]^ we propose that cancer cell clusters with an increasing amount of unjammed cells lead to decreased *stiffness* and increased *fluidity*. A recent study^[^ ^97^ ^]^ has shown that on the cellular level, the repeated mechanical deformations associated with tissue fluidization by unjamming trigger mechano‐protective mechanisms that lead to the formation of perinuclear actin rings and result in increased nucleus size and *stiffness* and furthermore, that due to the increased stress and strains unjammed cells are more prone to nuclear envelope rupture and consequential DNA damage, which further increases their malignancy. The unjammed cells showed also an upregulating of mesenchymal markers, e.g., ZEB1, which in turn is dependent on YAP/TAZ activity.^[^ ^97^ ^]^ The activation of YAP, the Yes‐associated protein and the highly related transcriptional coactivator with PDZ‐binding motif (TAZ) is deemed essential for the initiation or growth of most solid tumors.^[^ ^98^ ^]^ Since unjamming can occur both within^[^ ^21^ ^]^ and on the interface of clusters with the ECM,^[^ ^99^, ^100^ ^]^ these interactions between clusters and ECM may complicate the relationship between *fluidity* and *stiffness* further and lead to emergent effects that are discussed in more detail below. Our recent study with 1380 breast cancer patients proves that cancer cell unjamming significantly increases distant metastatic risk.^[^ ^18^ ^]^ Thus, we can be sure that the increased *fluidity* associated with unjamming and measured by MRE is a good prognostic parameter for tumor progression.

### 4.5 Tumor Microenvironment and Extracellular Matrix

The composition and architecture of the noncellular microenvironment can significantly affect a tissue's bulk mechanical properties.^[^ ^87^ ^]^ During tumor progression, the stroma can undergo structural changes^[^ ^101^ ^]^ that alter bulk *stiffness* and *fluidity*. In most solid tumors cancer cell clusters are embedded into a fibrotic extracellular matrix. The fibrosis leads to very dense and rigid ECM, which is expected to be main contributor why tumors are felt by palpation as a rigid mass. The fibrotic stroma in tumors is one of the most important mechanobiological tumor promoters that stimulates all hallmarks of cancer.^[^ ^102^ ^]^ Altered gene expression of cancer‐associated fibroblasts leads to a fibrotic accumulation of ECM components,^[^ ^103^ ^]^ resulting in collagen crosslinking.^[^ ^104^ ^]^ This increases the *stiffness* of the tumor environment^[^ ^105^ ^]^ and favors tumor progression.^[^ ^106^ ^]^ Crosslinking strengthens protein–protein interactions and leads to increased *stiffness* and decreased *fluidity* (as indicated by the green arrow in Figure 4). On the other hand, Tumor‐associated ECM remodeling is also affected by matrix degradation due to an abundance of matrix metalloproteinases,^[^ ^107^ ^]^ which can result in a loss of structural integrity, leading to increased viscous dissipation and therefore *fluidity* (as indicated by the red arrow in Figure 3c). A combination of both effects, fibrosis, and matrix degradation, can simultaneously increase *stiffness* and *fluidity*. Hepatocellular carcinoma (HCC), which is the third most common cause of cancer mortality^[^ ^2^ ^]^ occurs in most cases with a background of chronic liver disease or cirrhosis. Quantitative analysis of collagen concentrations found a 4–8 fold increase compared to healthy liver tissue.^[^ ^108^, ^109^ ^]^ Further alterations in structure and accumulation of dysmorphic collagen fibers are seen in malignant liver lesions,^[^ ^106^ ^]^ which further increase *stiffness* and *fluidity* in CCA and HCC, as seen in Figure 2. The prominent role of fibrosis is also confirmed in other solid tumors. The desmoplastic stroma of pancreatic tumors constitutes up to 80–85% of the tumor bulk.^[^ ^110^ ^]^ Rectal and prostate carcinomas also have a fibrotic background or are at least associated with a significant increase in collagen.^[^ ^38^, ^111^ ^]^ The increased *stiffness* and *fluidity* in MRE measurements are thus also a good indicator of fibrosis as a sign of rising tumor aggressiveness.

Collagen is the main structural ECM compartment of most systemic organs, but it is mostly absent in the healthy adult brain.^[^ ^112^ ^]^ Brain tissue is generally characterized as rather soft and recently even as superviscous.^[^ ^70^ ^]^ Between neurons the main constituents are different types of proteoglycans, heavily glycosylated proteins with one or several covalently attached glycosaminoglycan (GAG) chains.^[^ ^113^ ^]^ These GAGs are also part of the ECM throughout the whole body, filling the space between cells and forming large complexes with other proteoglycans, hyaluronic acid, or fibrous matrix components such as collagen.^[^ ^114^ ^]^ These large polar molecules can bind (i.e., catch away) large amounts of water. Through accumulation of GAGs, soft‐tissue ECM can turn a more liquid material into a more solid state.^[^ ^19^, ^115^ ^]^ An overexpression of GAGs will decrease *fluidity*, and conversely, chain cleavage or depletion of GAGs will cause an increase.^[^ ^115^, ^116^, ^117^ ^]^ As a result, a change in brain *fluidity* in either direction indicates disrupted homeostasis as a pathologic change.^[^ ^23^ ^]^ With respect to our brain data shown above, an abundance of GAGs was found in glioblastoma (GB) compared to meningioma (MEN) in a histopathological analysis.^[^ ^19^ ^]^ Collagen was only found in the bulk tissue of MEN. These tumors originate from the four membranes enveloping the brain (i.e., the collagen containing meninges) and do rather grow from there into the brain than in the brain, like the GB. This supports the in vivo MRE results that GB is softer but more solid‐like than MEN, as depicted in Figure 2. Additionally, our recent study found a correlation between single cell and bulk properties in GB but not in MEN,^[^ ^118^ ^]^ which could be another result of the lack of fibrous ECM in GB.

### 4.6 Emergent Effects of Cellular Unjamming in Specific Microenvironments

When unjammed cancer cells are in contact with their respective microenvironment emergent effects arise, which in our current understanding contribute further to the opposing mechanical bulk behavior of brain tumors and other solid tumors in the body. Tissue spreading behavior can be described as a competition or a *tug‐of‐war* ^[^ ^119^ ^]^ between cell‐cell and cell‐substrate adhesion. We have recently shown that when compared to nontumorous cells, cancer cells have a lower cortical contractility and a reduced ability to form collective actin rims around cellular clusters and therefore facilitating cell escape.^[^ ^56^ ^]^ The fibrotic collagen networks associated with body tumors enable strong cell‐ECM interactions, while those interactions are drastically reduced or even disabled in the brain microenvironment.

To be able to generate traction by adhesion and to move on a substrate, cells need a suitable environment with the correct anchor points to form focal adhesions. The resulting motility is closely linked to the formation of polarized actin stress fibers and the corresponding stress fiber‐mediated contractility. The ability to develop polarized stress fibers increases with substrate *stiffness*,^[^ ^120^, ^121^ ^]^ which in turn also increases cellular *stiffness*.^[^ ^122^ ^]^ While cells can also move on soft, more fluid substrates, cellular traction forces are still much higher on stiff, more solid substrates.^[^ ^123^ ^]^ Furthermore, cancer cells develop more polarized stress fibers compared to nontumorous cells.^[^ ^56^ ^]^ A recent study has also shown that under confinement stress fiber assembly is induced and cortical stiffness is reduced, which is also associated with a delocalization of YAP from the nucleus to the cytoplasm.^[^ ^124^ ^]^ Thus, increased cancer cell motility triggered by dipole‐like stress fiber‐mediated contractility manifests itself in an increased *stiffness* in MRE measurements. This also enables cancer cells to strongly pull on the ECM, which leads to long‐range, strain‐induced ECM stiffening. Evidence is provided in experiments where tumor spheroids on top of or embedded in a collagen gel were able to create large gel displacements,^[^ ^125^ ^]^ resulting in stiffening of the environment which further aligns the ECM fibers towards the cells and ultimately leads to invasion.^[^ ^56^ ^]^ All these microenvironment‐cancer cell interactions ultimately lead to increased tissue *stiffness*. With the accompanying increase in cellular motility, tumors also become more *fluid‐like*, accompanied by an increase in viscosity (*G*″) additionally contributing to the *stiffness* by changing |G*|. Since fibrotic ECM and cancer cells are in a positive feedback loop, we expect a strong impact towards higher *stiffness* and *fluidity*. Increased *fluidity* and *stiffness* as MRE signatures are thus also read outs of the mechanobiological feedback loop that acts a strong tumor promoter.

As argued in the paragraphs above we expect that tumor progression results in an increased *fluidity* and *stiffness* with respect to its MRE signature—when fibrous collagen structures are present. As the brain's environment lacks these structures, the interactions between tumor cells and the ECM in the brain are fundamentally different. We hypothesize that brain cancer cells’ ability to form stress fiber formation and exert traction forces are significantly reduced since collagen is missing as a substrate. Consequently, cellular unjamming in the brain ECM will more occur in the classical sense by a shift from an elastic to a viscous behavior, where *stiffness* decreases while *fluidity* increases. However, collagen is present in the basement membrane surrounding blood vessels^[^ ^19^ ^]^ and perivascular invasion has been discussed to be one of the drivers of GBs high spreading capacity.^[^ ^126^ ^]^ Combined with the overexpressing of GAGs and their intrinsic solidifying property to bind large amounts of water, this can explain why glioblastomas are more solid‐like and softer, while MEN that originate from the collagen‐containing meninges and lack the abundance of GAGs have higher tissue *fluidity* and *stiffness*. Furthermore, if the tumor environment itself is soft and viscous as in the superviscous brain, it can withstand little resistance against a growing tumor mass. Since GB does not build up a fibrous ECM and does not have to withstand strong deforming strains, compression stiffening is not fostered^[^ ^127^ ^]^ which will further contribute to the low *stiffness* of GBs. Nevertheless, in GBs tumor aggressiveness leads to distinctive signature with respect to *fluidity* and *stiffness* that can be detected in MRE measurements.

**Figure 6:**
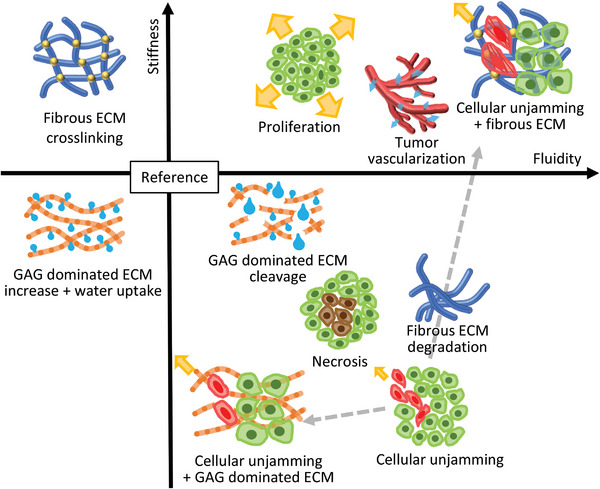
The influence of tumor‐associated mechanisms on bulk tissue *stiffness* and *fluidity* illustrated as shift along the *stiffness* or *fluidity* axis with respect to unaffected reference tissue. The mechanisms are listed in the same order as discussed in the text above: Tumor vascularization is associated with abundant and dysfunctional blood vessels, which can lead to the accumulation of “free” extracellular fluid that leads to a hydrodynamic pressure increase, resulting in increased tissue *fluidity* and *stiffness*. Necrosis leads to a loss of structural integrity, which decreases *stiffness* and increases *fluidity*. Proliferation represents cellular flows on long timescales leading to increased *fluidity*. The resulting strain leads to a pressure buildup, resulting in increased *stiffness*. Fibrosis and crosslinking of the ECM can drastically increase *stiffness* and reduce *fluidity*. While, enzymatic degradation of the ECM and dysmorphic accumulation of collagen, leads to increased *fluidity*. The ECM in the brain lacks collagen but has high amounts of long sugar chains, such as glycosaminoglycans (GAGs) that can bind large amounts of water and decrease *fluidity*. If GAGs are depleted or cleaved, it can increase *fluidity*. Cellular unjamming causes an increase in *fluidity* due to increased cell motility and a decrease in stiffness due to a reduction in elastic resistance. Associated emergent effects (grey dotted arrows) can be: cellular unjamming in cancer cell clusters embedded in a fibrous ECM result in both increased *stiffness* (due to the pulling of the cancer cells on the ECM enabling a positive feedback loop) and *fluidity* (due to increased motility). In contrast, cellular unjamming in a GAG‐dominated ECM does not allow for *stiffness* increase due to the lack of strong focal anchor points of the cells to collagen.

Although tumors exhibit a vast heterogeneity, the underlying biophysical mechanisms of tumor growth are probably universal and agnostic to many molecular details of different tumor entities. Indeed, our study has revealed a strikingly robust trend towards bulk tissue stiffening and fluidization in solid tumors. The effects of the above‐discussed mechanisms on bulk tissue *stiffness* and *fluidity* are condensed in **Figure** **6**. The trends that we observed in our meta‐analysis (see. Figure 2) and experiments (see Figures 3 and 4) are merged in our gedankenexperiment with respect to the macroscopic behavior on the tissue level. To describe the complex, multiscale compound mechanical behavior of tumor tissue on the bulk level, we used coarse graining to shift the scale away from molecular details and to reduce the parameters to defining the tumor properties to bulk *stiffness* and *fluidity* of sample and reference tissue, as well as *fluid heterogeneity* and the *tumor front texture* of the tumor front. The resulting roadmap towards a tumor classification is outlined in Figure 5. We demonstrated its applicability in various empirical cases for different solid tumor entities (see Table 3).

### 4.7 Limitations

Due to its limited resolution current clinical MRE can assess only the macroscopic bulk *stiffness* and *fluidity* of tumors. Their complementary implementation into MRI routines can improve clinical diagnosis.^[^ ^38^ ^]^ We presently cannot see all the mechanical details of a tumor in MRE. However, the coarse‐grained properties, *fluidity* and *stiffness*, that can be readily probed as MRE signature may have the advantage that the reductionist perspective in terms of the mechanical complexity of a solid tumor provides effective diagnostic parameters. Another limitation is that the pathologic, mechanical processes in tumors have characteristic time scales of hours to weeks that cannot be directly assessed by MRE. Nevertheless, the trends that we have found clearly show that these phenomena already manifest itself on the MRE relevant time scales. Moreover, MRE measurements are currently quantified by various material parameters, such a shear wave speed, storage and loss modulus etc. We see a strong need to unify data processing and imaging parameters and make them interchangeable. The introduction of data analysis tools that are openly available to the community, such as https://bioqic‐apps.charite.de/, which provide standardized markers of *stiffness* and *fluidity*, will promote the interchangeability of MRE parameters across clinical trials. Thus, we strive and hope that our results will trigger according clinical studies. For a more comprehensive prognosis and stratification of tumor progression analyzes of *fluid heterogeneity* and *tumor front texture* in individual tumors is necessary in most cases. These two factors have not been accessible in present clinical imaging, and it remains to be determined if this will be possible with current voxel edge sizes around 2 mm (see Table 1) in clinical scanners and millimeter thick slices in small animal setups.^[^ ^128^ ^]^ High magnetic field strength in MRI has recently allowed cellular resolution in tissue explants.^[^ ^129^ ^]^ If not in patients, this may have at least the potential for the analysis of 3D tissue mechanics in biopsies or a fast 3D analysis of tissue samples, which cannot be done by current histopathology relying on optical microscopy. As there is a clear trend towards higher imaging resolutions, parts of the resolution problem might be self‐resolving soon. MRE maps in the range of 1 mm^3^ voxel sizes should be attainable soon,^[^ ^130^ ^]^ which might be sufficient to establish tumor *heterogeneity* as a diagnostic factor. However, we do not anticipate that clinical MRE will reach a voxel size sufficient for adequate characterization of *tumor front texture* in the near future. A workaround for this resolution issue could be MRE's inherent sensitivity to wave refraction, even at sub‐voxel structures as small in the micrometer range.^[^ ^131^ ^]^ Also, changes in wave propagation characteristics at the interface between two tissue regions^[^ ^132^ ^]^ could provide further information on the *tumor front texture*, i.e., the mechanical connectivity of a tumor front to its surroundings as shown with slip interference imaging in meningioma.^[^ ^133^ ^]^ Information about the mechanical *heterogeneity* and *tumor front texture* of tumors may also be deduced from other MRI contrasts such as T1‐ and T2 mapping sequences with the help of artificial neuronal networks.^[^ ^134^, ^135^ ^]^ In any case, MRE based on *fluidity* and *stiffness* have the translational potential for a diagnostic improvement.

### 4.8 Outlook/Conclusion

State of the art tumor diagnosis relies in its staging, grading, and stratification predominately on histopathology and more recently on molecular tumor markers such as gene signatures, which requires an invasive biopsy or tumorectomy. While radiological imaging is mostly used to detect and locate a tumor. For many tumor entities biopsies are difficult to take and probe only a small cut out from the tumor. Moreover, with advancing oncology an increasing amount of therapy decisions have to be made before surgical removal of the tumor, which increase the need for noninvasive tumor markers that transform radiology.

MRE‐based measurements of tissue *stiffness* and *fluidity* as novel biomechanical tumor markers are a paradigm shift in more than one sense. Instead of a molecular predictor we apply a biomechanical signature as marker and we use MRE not just as an imaging contrast, but as a functional readout of tumor progression. Previous MRE has focused on *stiffness*. Tissue *fluidity* is a novel MRE parameter that provides a better contrast than *stiffness* alone and most importantly is a prognostic marker for cancer cell unjamming as an early event of the metastatic cascade, increased proliferation, and vascularization. The combination with *stiffness* provides reliable trends that prognoses increasing tumor aggressiveness induced by increased cell proliferation, early metastatic events in the primary tumor before the cancer cells have left the tumor, fibrosis including mechanobiological feedback, and vascularization. We would like to stress that *fluidity* is sensitive for cell motility, a cancer cell function where otherwise no good markers exist besides the lymph node status, which only becomes relevant when the cancer cells have already left the tumor. *Fluidity* and *stiffness* are coarse grained pathologic mechanical properties that we can relate to several cancer drivers, hallmarks of cancer, to evaluate cancer risk without cellular resolution. The connection between pathologic mechanical changes and tumor invasion that we establish here will push MRE beyond an imaging technique towards a functional stratification tool capable of monitoring and predicting the potential for aggressive growth and metastatic spread. The *displacing growth* mode we described in our gedankenexperiment is concerned with a tumor's ability to push away surrounding tissue. Analyzing tissue *stiffness* and *fluidity* after surgical removal and/or as a monitoring step during radiation therapy could provide vital information about the success and progress of the treatment since it should stop displacing growth. As remaining cancer cells are linked to relapse and thus clinical outcomes, the *fluidity* signature of the excision rim should have a high predictive power for long‐term treatment success. Complete tumorectomy, which can involve removing large areas of surrounding tissue, is often not feasible, detailed maps of tissue *fluidity* and *tumor front texture* could help guide surgeons and radiation oncologists to refine the definition of resection and treatment margins.^[^ ^136^ ^]^ In general, tissue *fluidity* as a marker could directly detect and monitor a tumor's responsiveness to treatment. Naturally, it will need profound clinical studies that relate MRE‐data with patient outcome to determine the prognostic and diagnostic value of our proposed mechanics‐based stratification scheme and with the expected progress in MRE tumor *heterogeneity* and *tumor front texture* will refine our prognostic approach.

We do not see our proposed stratification scheme as an alternative to established cancer diagnosis. We hope that our novel biomechanical markers can be used complementary to already established methods. They may significantly add to standard clinical imaging workflow, as already existing MRI hardware can be readily upgraded. With our suggested approach MRE can deliver new tumor markers that can be easily implemented at affordable costs. They do not require invasively obtained tissue samples, ionizing radiation or contrast agents, and thus are perfectly suited for early prognostic diagnosis and comprehensive therapy monitoring. Thus, our approach may help personalized medicine aiming towards more individualized or adaptive tumor therapies^[^ ^137^ ^]^ and reducing the risk of over‐ and undertreatment. In synopsis, while MRE has been previously used for imaging using *stiffness* alterations as a contrast mechanism to detect density differences in tissues, we suggest to use pathological biomechanical changes in solid tumors as an efficient readout of tumor progression.

## Conflict of Interest

The authors declare no conflict of interest.

## Supporting information

Supporting Information

Supplemental Video 1

Supplemental Video 2

Supplemental Video 3

Supplemental Video 4

## Data Availability

The data that support the findings of this study are available from the corresponding author upon reasonable request.

## References

[1] Global health estimates: Leading causes of death, https://www.who.int/data/gho/data/themes/mortality‐and‐global‐health‐estimates/ghe‐leading‐causes‐of‐death (accessed: June, 2021).

[2] H. Sung , J. Ferlay , R. L. Siegel , M. Laversanne , I. Soerjomataram , A. Jemal , F. Bray , Ca: Cancer J. Clin. 2021, 71, 209. 33538338 10.3322/caac.21660

[3] E. Dolgin , Nature 2018, 555, S26. 10.1038/d41586-018-02483-3 32095016

[4] A to Z List of Cancer Types ‐ National Cancer Institute. [Online]. Available: https://www.cancer.gov/types (accessed: June, 2021).

[5] N. Harbeck , F. Penault‐Llorca , J. Cortes , M. Gnant , N. Houssami , P. Poortmans , K. Ruddy , J. Tsang , F. Cardoso , Nat. Rev. Dis. Primers 2019, 5, 1. 31548545 10.1038/s41572-019-0111-2

[6] Z. Varga , P. Sinn , F. Fritzsche , A. Von Hochstetter , A. Noske , P. Schraml , C. Tausch , A. Trojan , H. Moch , PLoS One 2013, 8, e58483. 23505515 10.1371/journal.pone.0058483 PMC3591350

[7] I. Sestak , R. Buus , J. Cuzick , P. Dubsky , R. Kronenwett , C. Denkert , S. Ferree , D. Sgroi , C. Schnabel , F. L. Baehner , E. Mallon , M. Dowsett , JAMA Oncol. 2018, 4, 545. 29450494 10.1001/jamaoncol.2017.5524 PMC5885222

[8] J. S. Boehm , M. J. Garnett , D. J. Adams , H. E. Francies , T. R. Golub , W. C. Hahn , F. Iorio , J. M. McFarland , L. Parts , F. Vazquez , Nature 2021, 589, 514. 33500573 10.1038/d41586-021-00182-0

[9] E. Jonietz , Nature 2012, 491, S56. 23320288 10.1038/491s56a

[10] K. R. Fischer , A. Durrans , S. Lee , J. Sheng , F. Li , S. T. C. Wong , H. Choi , T. El Rayes , S. Ryu , J. Troeger , R. F. Schwabe , L. T. Vahdat , N. K. Altorki , V. Mittal , D. Gao , Nature 2015, 527, 472. 26560033 10.1038/nature15748 PMC4662610

[11] X. Ye , T. Brabletz , Y. Kang , G. D. Longmore , A. M. Nieto , B. Z. Stanger , J. Yang , R. A. Weinberg , Nature 2017, 547, E1. 28682326 10.1038/nature22816 PMC6283276

[12] M. K. Jolly , S. A. Mani , H. Levine , Biochim. Biophys. acta. Rev. Cancer 2018, 1870, 151. 29997040 10.1016/j.bbcan.2018.07.001

[13] M. K. Jolly , K. E. Ware , S. Gilja , J. A. Somarelli , H. Levine , Mol. Oncol. 2017, 11, 755. 28548345 10.1002/1878-0261.12083 PMC5496498

[14] P. Chakraborty , J. T. George , S. Tripathi , H. Levine , M. K. Jolly , Front. Bioeng. Biotechnol. 2020, 8, 220. 32266244 10.3389/fbioe.2020.00220 PMC7100584

[15] V. Padmanaban , I. Krol , Y. Suhail , B. M. Szczerba , N. Aceto , J. S. Bader , A. J. Ewald , Nature 2019, 573, 439. 31485072 10.1038/s41586-019-1526-3 PMC7365572

[16] W. R. Dawson , B. Ebbell , J. Egypt Archaeol. 1938, 24, 250.

[17] T. Fuhs , F. Wetzel , A. W. Fritsch , X. Li , R. Stange , S. Pawlizak , T. R. Kießling , E. Morawetz , S. Grosser , F. Sauer , J. Lippoldt , F. Renner , S. Friebe , M. Zink , K. Bendrat , J. Braun , M. H. Oktay , J. Condeelis , S. Briest , B. Wolf , L‐C. Horn , M. Höckel , B. Aktas , M. C. Marchetti , M. L. Manning , A. Niendorf , D. Bi , J. A. Käs , Nat. Phys. 2022, 18, 1510.

[18] P. Gottheil , J. Lippoldt , S. Grosser , F. Renner , M. Saibah , D. Tschodu , A.‐K. Poßögel , A.‐S. Wegscheider , B. Ulm , K. Friedrichs , C. Lindner , C. Engel , M. Löffler , B. Wolf , M. Höckel , B. Aktas , H. Kubitschke , A. Niendorf , J. A. Käs , Phys. Rev. X 2023, 13, 031003.

[19] K.‐J. Streitberger , L. Lilaj , F. Schrank , J. Braun , K. T. Hoffmann , M. Reiss‐Zimmermann , J. A. Käs , I. Sack , Proc. Natl. Acad. Sci. USA 2020, 217, 128. 10.1073/pnas.1913511116 PMC6955323 31843897

[20] A. Fritsch , M. Höckel , T. Kiessling , K. D. Nnetu , F. Wetzel , M. Zink , J. A. Käs , Nat. Phys. 2010, 6, 730.

[21] S. Grosser , J. Lippoldt , L. Oswald , M. Merkel , D. M. Sussman , F. Renner , P. Gottheil , E. W. Morawetz , T. Fuhs , X. Xie , S. Pawlizak , A. W. Fritsch , B. Wolf , L.‐C. Horn , S. Briest , B. Aktas , L. M. Manning , J. A. Käs , Phys. Rev. X 2021, 11, 011033.

[22] J. J. Fredberg , Soft Matter 2022, 18, 2346. 35244652 10.1039/d1sm01716k PMC8957601

[23] D. Hanahan , R. A. Weinberg , Cell 2011, 144, 646. 21376230 10.1016/j.cell.2011.02.013

[24] D. Bi , J. H. Lopez , J. M. Schwarz , M. L. Manning , Nat. Phys. 2015, 11, 1074.

[25] O. Ilina , P. G. Gritsenko , S. Syga , J. Lippoldt , C. A. M. La Porta , O. Chepizhko , S. Grosser , M. Vullings , G.‐J. Bakker , J. Starruß , P. Bult , S. Zapperi , J. A. Käs , A. Deutsch , P. Friedl , Nat. Cell Biol. 2020, 22, 1103. 32839548 10.1038/s41556-020-0552-6 PMC7502685

[26] J.‐A. Park , J. H. Kim , D. Bi , J. A. Mitchel , N. T. Qazvini , K. Tantisira , C. Y. Park , M. Mcgill , S.‐H. Kim , B. Gweon , J. Notbohm , R. Steward Jr , S. Burger , S. H. Randell , A. T. Kho , D. T. Tambe , C. Hardin , S. A. Shore , E. Israel , D. A. Weitz , D. J. Tschumperlin , E. P. Henske , S. T. Weiss , M. L. Manning , J. P. Butler , J. M. Drazen , J. J. Fredberg , Nat. Mater. 2015, 14, 1040. 26237129 10.1038/nmat4357 PMC4666305

[27] J.‐A. Park , L. Atia , J. A. Mitchel , J. J. Fredberg , J. P. Butler , J. Cell Sci. 2016, 129, 3375. 27550520 10.1242/jcs.187922 PMC5047682

[28] P. Friedl , J. Locker , E. Sahai , J. E. Segall , Nat. Cell Biol. 2012, 14, 777. 22854810 10.1038/ncb2548

[29] P. Friedl , D. Gilmour , Nat. Rev. Mol. Cell Biol. 2009, 10, 445. 19546857 10.1038/nrm2720

[30] L. Oswald , S. Grosser , D. M. Smith , J. A. Käs , J. Phys. D: Appl. Phys. 2017, 50, 483001. 29628530 10.1088/1361-6463/aa8e83 PMC5884432

[31] E. Blauth , H. Kubitschke , P. Gottheil , S. Grosser , J. A. Käs , Front. Phys. 2021, 9, 445.

[32] H. T. Nia , H. Liu , G. Seano , M. Datta , D. Jones , N. Rahbari , J. Incio , V. P. Chauhan , K. Jung , J. D. Martin , V. Askoxylakis , T. P. Padera , D. Fukumura , Y. Boucher , F. J. Hornicek , A. J. Grodzinsky , J. W. Baish , L. L. Munn , R. K. Jain , Nat. Biomed. Eng. 2016, 1, 0004. 28966873 10.1038/s41551-016-0004 PMC5621647

[33] H. T. Nia , M. Datta , G. Seano , P. Huang , L. L. Munn , R. K. Jain , Nat. Protoc. 2018, 13, 1091. 29674756 10.1038/nprot.2018.020 PMC6546092

[34] S. Hirsch , J. Braun , I. Sack , Magnetic Resonance Elastography – Physical Background and Medical Applications 2017, http://doi.wiley.com/10.1002/9783527696017 (accessed: January, 2021).

[35] S. Papazoglou , S. Hirsch , J. Braun , I. Sack , Phys. Med. Biol. 2012, 57, 2329. 22460134 10.1088/0031-9155/57/8/2329

[36] Y. Liu , M. Wang , R. Ji , L. Cang , F. Gao , Y. Shi , Clin. Radiol. 2018, 73, 865. 29895389 10.1016/j.crad.2018.05.016

[37] C. Balleyguier , A. B. Lakhdar , A. Dunant , M.‐C. Mathieu , S. Delaloge , R. Sinkus , NMR Biomed. 2018, 31, e3795. 10.1002/nbm.3795 29073719

[38] M. Li , J. Guo , P. Hu , H. Jiang , J. Chen , J. Hu , P. Asbach , I. Sack , W. Li , Radiology 2021, 299, 362. 33687285 10.1148/radiol.2021201852

[39] J. Hu , J. Guo , Y. Pei , P. Hu , M. Li , I. Sack , W. Li , Front. Oncol. 2021, 11, 3201. 10.3389/fonc.2021.701336 PMC8415020 34485136

[40] Y. Wang , J. Guo , D. Ma , J. Zhou , Y. Yang , Y. Chen , H. Wang , I. Sack , R. Li , F. Yan , Front. Oncol. 2022, 12, 962272. 36518314 10.3389/fonc.2022.962272 PMC9744252

[41] X. Hu , J. Zhou , Y. Li , Y. Wang , J. Guo , I. Sack , W. Chen , F. Yan , R. Li , C. Wang , Cancers 2022, 14, 2575. 35681558 10.3390/cancers14112575 PMC9179448

[42] M. Shahryari , H. Tzschätzsch , J. Guo , S. R. Marticorena Garcia , G. Böning , U. Fehrenbach , L. Stencel , P. Asbach , B. Hamm , J. A. Käs , J. Braun , T. Denecke , I. Sack , Cancer Res. 2019, 79, 5704. 31551364 10.1158/0008-5472.CAN-19-2150

[43] L. Zhu , J. Guo , Z. Jin , H. Xue , M. Dai , W. Zhang , Z. Sun , J. Xu , S. R. Garcia Marticorena , P. Asbach , B. Hamm , I. Sack , Eur. Radiol. 2020, 31, 3366. 33125553 10.1007/s00330-020-07420-5

[44] S. R. Marticorena Garcia , L. Zhu , E. Gültekin , R. Schmuck , C. Burkhardt , M. Bahra , D. Geisel , M. Shahryari , J. Braun , B. Hamm , Z.‐Y.u Jin , I. Sack , J. Guo , Invest Radiol 2020, 55, 769. 32796197 10.1097/RLI.0000000000000704

[45] P. Asbach , S.‐R. Ro , N. Aldoj , J. Snellings , R. Reiter , J. Lenk , T. Köhlitz , M. Haas , J. Guo , B. Hamm , J. Braun , I. Sack , Invest. Radiol. 2020, 55, 524. 32496317 10.1097/RLI.0000000000000685

[46] S. R. Mousavi , A. Fehlner , K.‐J. Streitberger , J. Braun , A. Samani , I. Sack , J. Biomech. 2014, 47, 1652. 24656483 10.1016/j.jbiomech.2014.02.038

[47] H. Tzschätzsch , J. Guo , F. Dittmann , S. Hirsch , E. Barnhill , K. Jöhrens , J. Braun , I. Sack , Med. Image Anal. 2016, 30, 1. 26845371 10.1016/j.media.2016.01.001

[48] Density » IT'IS Foundation , https://itis.swiss/virtual‐population/tissue‐properties/database/density/ (accessed: July 2021).

[49] J. Guo , L. Savic , K. Hillebrandt , I. Sack , Invest. Radiol. 2023, 58, 578. 36897804 10.1097/RLI.0000000000000971

[50] J. Braun , H. Tzschätzsch , C. Körting , A. Ariza De Schellenberger , M. Jenderka , T. Drießle , M. Ledwig , I. Sack , Magn. Reson. Med. 2018, 79, 470. 28321914 10.1002/mrm.26659

[51] D. Klatt , C. Friedrich , Y. Korth , R. Vogt , J. Braun , I. Sack , Biorheology 2010, 47, 133. 20683156 10.3233/BIR-2010-0565

[52] V. D. Djordjevic , J. Jaric , B. Fabry , J. J. Fredberg , D. Stamenovic , Ann. Biomed. Eng. 2003, 31, 692. 12797619 10.1114/1.1574026

[53] F. Sauer , L. Oswald , A. Ariza De Schellenberger , H. Tzschätzsch , F. Schrank , T. Fischer , J. Braun , C. T. Mierke , R. Valiullin , I. Sack , J. A. Käs , Soft Matter 2019, 15, 3055. 30912548 10.1039/c8sm02264j

[54] M. Sadati , N. Taheri Qazvini , R. Krishnan , C. Y. Park , J. J. Fredberg , Differentiation 2013, 86, 121. 23791490 10.1016/j.diff.2013.02.005 PMC3795803

[55] A. Haeger , M. Krause , K. Wolf , P. Friedl , Biochim. Biophys. Acta – Gen. Subj. 2014, 1840, 2386. 10.1016/j.bbagen.2014.03.020 24721714

[56] E. Warmt , S. Grosser , E. Blauth , X. Xie , H. Kubitschke , R. Stange , F. Sauer , J. Schnauß , J. M. Tomm , M. Von Bergen , J. A. Käs , New J. Phys. 2021, 23, 103020.

[57] A. Hayn , T. Fischer , C. T. Mierke , Front. Cell Dev. Biol. 2020, 8, 583226. 33043017 10.3389/fcell.2020.583226 PMC7527720

[58] R. Reiter , M. Shahryari , H. Tzschätzsch , M. Haas , C. Bayerl , B. Siegmund , B. Hamm , P. Asbach , J. Braun , I. Sack , J. Mech. Behav. Biomed. Mater. 2021, 121, 104645. 34166871 10.1016/j.jmbbm.2021.104645

[59] A. Mongera , P. Rowghanian , H. J. Gustafson , E. Shelton , D. A. Kealhofer , E. K. Carn , F. Serwane , A. A. Lucio , J. Giammona , O. Campàs , Nature 2018, 561, 401. 30185907 10.1038/s41586-018-0479-2 PMC6148385

[60] J. Ranft , M. Basan , J. Elgeti , J.‐F. Joanny , J. Prost , F. Jülicher , Proc. Natl. Acad. Sci. USA 2010, 107, 20863. 21078958 10.1073/pnas.1011086107 PMC3000289

[61] M. J. Bogdan , T. Savin , R. Soc. Open Sci. 2018, 5, 181579. 30662758 10.1098/rsos.181579 PMC6304124

[62] C. J. Chan , A. E. Ekpenyong , S. Golfier , W. Li , K. J. Chalut , O. Otto , J. Elgeti , J. Guck , F. Lautenschläger , Biophys. J. 2015, 108, 1856. 25902426 10.1016/j.bpj.2015.03.009 PMC4407259

[63] Z. Wang , F. Wang , Y.i Peng , Z. Zheng , Y. Han , Science 2012, 338, 87. 23042889 10.1126/science.1224763

[64] P. Friedl , K. Wolf , Nat. Rev. Cancer 2003, 3, 362. 12724734 10.1038/nrc1075

[65] I. Dagogo‐Jack , A. T. Shaw , Nat. Rev. Clin. Oncol. 2018, 15, 81. 29115304 10.1038/nrclinonc.2017.166

[66] J. Zhou , Z. Tang , S. Gao , C. Li , Y. Feng , X. Zhou , Front Oncol 2020, 10, 188. 32161718 10.3389/fonc.2020.00188 PMC7052362

[67] P. B. Georgieva , T. Mathivet , S. Alt , W. Giese , M. Riva , M. Balcer , H. Gerhardt , Neuro‐Oncol. Adv. 2020, 2. 10.1093/noajnl/vdaa127 PMC7649962 33205045

[68] C. Morrison , Nat. Rev. Drug Discovery 2016, 15, 373. 27245386 10.1038/nrd.2016.111

[69] M. J. Paszek , N. Zahir , K. R. Johnson , J. N. Lakins , G. I. Rozenberg , A. Gefen , C. A. Reinhart‐King , S. S. Margulies , M. Dembo , D. Boettiger , D. A. Hammer , V. M. Weaver , Cancer Cell 2005, 8, 241. 16169468 10.1016/j.ccr.2005.08.010

[70] H. Herthum , S. C. H. Dempsey , A. Samani , F. Schrank , M. Shahryari , C. Warmuth , H. Tzschätzsch , J. Braun , I. Sack , Acta Biomater. 2021, 121, 393. 33326885 10.1016/j.actbio.2020.12.027

[71] H. M. Phillips , M. S. Steinberg , Proc. Natl. Acad. Sci. USA 1969, 64, 121.5262993

[72] J. D. Amack , M. L. Manning , Science 2012, 338, 212. 23066072 10.1126/science.1223953

[73] D. J. Höppener , P. M. H. Nierop , E. Herpel , N. N. Rahbari , M. Doukas , P. B. Vermeulen , D. J. Grünhagen , C. Verhoef , Clin. Exp. Metastasis 2019, 36, 311. 31134394 10.1007/s10585-019-09975-0 PMC6611753

[74] L.‐C. Horn , U. Fischer , G. Raptis , K. Bilek , B. Hentschel , C. E. Richter , U.‐D. Braumann , J. Einenkel , Gynecol. Oncol. 2006, 103, 906. 16876852 10.1016/j.ygyno.2006.05.046

[75] J. Einenkel , U.‐D. Braumann , L.‐C. Horn , N. Pannicke , J.‐P. Kuska , A. Schütz , B. Hentschel , M. Höckel , Comput. Med. Imaging Graph 2007, 31, 428. 17521881 10.1016/j.compmedimag.2007.03.004

[76] L.‐C. Horn , C. E. Richter , B. Hentschel , A. Schütz , H. Pilch , C. Leo , M. Höckel , Ann. Diagn. Pathol. 2006, 10, 253. 16979515 10.1016/j.anndiagpath.2005.09.003

[77] E. G. McFarland , W. W. Mayo‐Smith , S. Saini , P. F. Hahn , M. A. Goldberg , M. J. Lee , Radiology 1994, 193, 43. 8090920 10.1148/radiology.193.1.8090920

[78] K. Miles , Br. J. Cancer 2020, 122, 929. 31937924 10.1038/s41416-019-0699-8 PMC7109132

[79] P. Carmeliet , R. K. Jain , Nature 2000, 407, 249. 11001068 10.1038/35025220

[80] A. R. Pries , M. Höpfner , F. Le Noble , M. W. Dewhirst , T. W. Secomb , Nat. Rev. Cancer 2010, 10, 587. 20631803 10.1038/nrc2895 PMC3109666

[81] H. Hashizume , P. Baluk , S. Morikawa , J. W. Mclean , G. Thurston , S. Roberge , R. K. Jain , D. M. Mcdonald , Am. J. Pathol. 2000, 156, 1363. 10751361 10.1016/S0002-9440(10)65006-7 PMC1876882

[82] J. M. Dunleavey , A. C. Dudley , Curr. Angiogenes 2012, 133, 133. 10.2174/2211552811201020133 PMC3982611 24729954

[83] W. Douglas Thompson , J. Pathol. 2001, 193, 425. 11275999 10.1002/1096-9896(200104)193:4<425::AID-PATH830>3.0.CO;2-E

[84] T. Hompland , K. V. Lund , C. Ellingsen , G. B. Kristensen , E. K. Rofstad , Radiother. Oncol. 2014, 113, 132. 25443501 10.1016/j.radonc.2014.09.011

[85] C.‐H. Heldin , K. Rubin , K. Pietras , A. Östman , Nat. Rev. Cancer 2004, 4, 806. 15510161 10.1038/nrc1456

[86] S. Y. Lee , M. K. Ju , H. M. Jeon , E. K. Jeong , Y. J. Lee , C. H. Kim , H. G. Park , S. I. Han , H. S. Kang , Oxid. Med. Cell Longev. 2018, 2018, 1. 10.1155/2018/3537471 PMC5831895 29636841

[87] J. Li , K. Zormpas‐Petridis , J. K. R. Boult , E. L. Reeves , A. Heindl , M. Vinci , F. Lopes , C. Cummings , C. J. Springer , L. Chesler , C. Jones , J. C. Bamber , Y. Yuan , R. Sinkus , Y. Jamin , S. P. Robinson , Cancer Res. 2019, 79, 5874. 31604713 10.1158/0008-5472.CAN-19-1595

[88] E. Lawson‐Keister , M. L. Manning , Curr. Opin. Cell Biol. 2021, 72, 146. 34461581 10.1016/j.ceb.2021.07.011

[89] P. Katira , M. H. Zaman , R. T. Bonnecaze , Phys. Rev. Lett. 2012, 108, 028103. 22324713 10.1103/PhysRevLett.108.028103

[90] G. A. Reddy , P. Katira , Soft Matter 2022, 18, 3713. 35502875 10.1039/d2sm00174h

[91] M. Saadaoui , D. Rocancourt , J. Roussel , F. Corson , J. Gros , Science 2020, 367, 453. 31974255 10.1126/science.aaw1965

[92] M. Czajkowski , D. M. Sussman , M. C. Marchetti , M. L. Manning , Soft Matter 2019, 15, 9133. 31674622 10.1039/c9sm00916g

[93] N. Schierbaum , J. Rheinlaender , T. E. Schäffer , Acta Biomater. 2017, 55, 239. 28396292 10.1016/j.actbio.2017.04.006

[94] J. A. Mitchel , A. Das , M. J. O'Sullivan , I. T. Stancil , S. J. DeCamp , S. Koehler , O. H. Ocaña , J. P. Butler , J. J. Fredberg , A. M. Nieto , D. Bi , J.‐A. Park , Nat. Commun. 2020, 11, 5053. 33028821 10.1038/s41467-020-18841-7 PMC7542457

[95] A. Saraswathibhatla , J. Notbohm , Phys. Rev. X 2020, 10, 011016.

[96] D. M. Sussman , M. Merkel , Soft Matter 2018, 14, 3397. 29667689 10.1039/c7sm02127e

[97] E. Frittoli , A. Palamidessi , F. Iannelli , F. Zanardi , S. Villa , L. Barzaghi , H. Abdo , V. Cancila , G. V. Beznoussenko , G. Chiar Della , M. Pagani , C. Malinverno , D. Bhattacharya , F. Pisati , W. Yu , V. Galimberti , G. Bonizzi , E. Martini , A. A. Mironov , U. Gioia , F. Ascione , Q. Li , K. Havas , S. Magni , Z. Lavagnino , F. A. Pennacchio , P. Maiuri , S. Caponi , M. Mattarelli , S. Martino , et al., Nat Mater. 2022, 22, 644. 36581770 10.1038/s41563-022-01431-x PMC10156599

[98] F. Zanconato , M. Cordenonsi , S. Piccolo , Cancer Cell 2016, 29, 783. 27300434 10.1016/j.ccell.2016.05.005 PMC6186419

[99] Y. L. Han , A. F. Pegoraro , H. Li , K. Li , Y. Yuan , G. Xu , Z. Gu , J. Sun , Y. Hao , S. K. Gupta , Y. Li , W. Tang , H. Kang , L. Teng , J. J. Fredberg , M. Guo , Nat. Phys. 2020, 16, 101. 32905405 10.1038/s41567-019-0680-8 PMC7469976

[100] A. Palamidessi , C. Malinverno , E. Frittoli , S. Corallino , E. Barbieri , S. Sigismund , G. V. Beznoussenko , E. Martini , M. Garre , I. Ferrara , C. Tripodo , F. Ascione , E. A. Cavalcanti‐Adam , Q. Li , P. P. Di Fiore , D. Parazzoli , F. Giavazzi , R. Cerbino , G. Scita , Nat. Mater. 2019, 18, 1252. 31332337 10.1038/s41563-019-0425-1

[101] N. E. Sounni , A. Noel , Clin. Chem. 2013, 59, 85. 23193058 10.1373/clinchem.2012.185363

[102] M. W. Pickup , J. K. Mouw , V. M. Weaver , EMBO Rep. 2014, 15, 1243. 25381661 10.15252/embr.201439246 PMC4264927

[103] G. S. Karagiannis , T. Poutahidis , S. E. Erdman , R. Kirsch , R. H. Riddell , E. P. Diamandis , Mol. Cancer Res. 2012, 10, 1403. 23024188 10.1158/1541-7786.MCR-12-0307 PMC4399759

[104] T. R. Cox , D. Bird , A.‐M. Baker , H. E. Barker , M. W. Y. Ho , G. Lang , J. T. Erler , Cancer Res. 2013, 73, 1721. 23345161 10.1158/0008-5472.CAN-12-2233 PMC3672851

[105] C. T. Mierke , Physics of Cancer, IOP Publishing, 2018.

[106] K. R. Levental , H. Yu , L. Kass , J. N. Lakins , M. Egeblad , J. T. Erler , S. F. T. Fong , K. Csiszar , A. Giaccia , W. Weninger , M. Yamauchi , D. L. Gasser , V. M. Weaver , Cell 2009, 139, 891. 19931152 10.1016/j.cell.2009.10.027 PMC2788004

[107] S. Jodele , L. Blavier , J. M. Yoon , Y. A. Declerck , Cancer Metastasis Rev. 2006, 25, 35. 16680570 10.1007/s10555-006-7887-8

[108] M. Rojkind , M.‐A. Giambrone , L. Biempica , Gastroenterology 1979, 76, 710. 421999

[109] J. Gu , E. Zhang , B. Liang , Z. Zhang , X. Chen , M. Xiong , Z. Huang , Ann. Surg. Oncol. 2021, 28, 4227. 33452603 10.1245/s10434-020-09557-5

[110] A. Bulle , K. H. Lim , Signal. Transduct. Target Ther. 2020, 5, 249. 33122631 10.1038/s41392-020-00341-1 PMC7596088

[111] N. Yanagisawa , R. Li , D. Rowley , H. Liu , D. Kadmon , B. J. Miles , T. M. Wheeler , G. E. Ayala , Hum. Pathol. 2007, 38, 1611. 17868773 10.1016/j.humpath.2007.04.008

[112] D. Bonneh‐Barkay , C. A. Wiley , Brain Pathol. 2009, 19, 573. 18662234 10.1111/j.1750-3639.2008.00195.x PMC2742568

[113] G. Meisenberg , W. H. (Medical scientist) Simmons 2006, 678.

[114] D. Voet , J. G. Voet , C. W. Pratt , in Fundamentals of Biochemistry: Life at the Molecular Level, Wiley, USA 2016, 1184.

[115] P. A. Netti , D. A. Berk , M. A. Swartz , A. J. Grodzinsky , R. K. Jain , Cancer Res. 2000, 60, 2497. 10811131

[116] A. Ariza de Schellenberger , J. Bergs , I. Sack , M. Taupitz , in Quantification of Biophysical Parameters in Medical Imaging,Springer International Publishing, Cham 2018, pp. 123–150.

[117] K. Kohyama , Y. Sano , E. Doi , J. Agric. Food Chem. 1995, 43, 1808.

[118] F. Sauer , A. Fritsch , S. Grosser , S. Pawlizak , T. Kießling , M. Reiss‐Zimmermann , M. Shahryari , W. C. Müller , K.‐T. Hoffmann , J. A. Käs , I. Sack , Soft Matter 2021, 17, 10744. 34787626 10.1039/d1sm01291f PMC9386686

[119] P. L. Ryan , R. A. Foty , J. Kohn , M. S. Steinberg , Proc. Natl. Acad. Sci. USA 2001, 98, 4323. 11274361 10.1073/pnas.071615398 PMC31833

[120] M. Prager‐Khoutorsky , A. Lichtenstein , R. Krishnan , K. Rajendran , A. Mayo , Z. Kam , B. Geiger , A. D. Bershadsky , Nat. Cell Biol. 2011, 13, 1457. 22081092 10.1038/ncb2370

[121] M. Gupta , Bibhu Ranjan Sarangi , J. Deschamps , Y. Nematbakhsh , A. Callan‐Jones , F. Margadant , R. Mège , C. T. Lim , R. Voituriez , B. Ladoux , Nat. Commun. 2015, 6, 7525. 26109233 10.1038/ncomms8525 PMC4599139

[122] N. Gavara , R. S. Chadwick , Biomech. Model. Mechanobiol. 2016, 15, 511. 26206449 10.1007/s10237-015-0706-9 PMC4869747

[123] K. Mandal , D. Raz‐Ben Aroush , Z. T. Graber , B. Wu , C. Y. Park , J. J. Fredberg , W. Guo , T. Baumgart , P. A. Janmey , ACS Nano 2019, 13, 203. 30500159 10.1021/acsnano.8b05286 PMC6511072

[124] C. Rianna , M. Radmacher , S. Kumar , Mol. Biol. Cell 2020, 31, 1726. 31995446 10.1091/mbc.E19-10-0588 PMC7521845

[125] C. Mark , T. J. Grundy , P. L. Strissel , D. Böhringer , N. Grummel , R. Gerum , J. Steinwachs , C. C. Hack , M. W. Beckmann , M. Eckstein , R. Strick , G. M. O'Neill , B. Fabry , Elife 2020, 9. 10.7554/eLife.59538 PMC7266637 32484778

[126] S. Pacioni , Q. G. D'alessandris , M. Buccarelli , A. Boe , M. Martini , L. M. Larocca , G. Bolasco , L. Ricci‐Vitiani , M. L. Falchetti , R. Pallini , Cancers 2019, 12, 18. 31861603 10.3390/cancers12010018 PMC7017006

[127] J. L. Shivers , J. Feng , A. S. G. Van Oosten , H. Levine , P. A. Janmey , F. C. Mackintosh , Proc. Natl. Acad. Sci. USA 2020, 117, 21037. 32817547 10.1073/pnas.2003037117 PMC7474641

[128] G. Bertalan , J. Guo , H. Tzschätzsch , C. Klein , E. Barnhill , I. Sack , J. Braun , Magn. Reson. Med. 2019, 81, 2676. 30393887 10.1002/mrm.27586

[129] R. Van Schadewijk , J. R. Krug , D. Shen , K. B. S. Sankar Gupta , F. J. Vergeldt , T. Bisseling , A. G. Webb , H. Van As , A. H. Velders , H. J. M. De Groot , A. Alia , Sci. Rep. 2020, 10, 971. 31969628 10.1038/s41598-020-57861-7 PMC6976659

[130] J. Braun , J. Guo , R. Lützkendorf , J. Stadler , S. Papazoglou , S. Hirsch , I. Sack , J. Bernarding , NeuroImage 2014, 90, 308. 24368262 10.1016/j.neuroimage.2013.12.032

[131] I. Sack , Nat. Rev. Phys. 2022, 5, 25.

[132] S. Papazoglou , U. Hamhaber , J. Braun , I. Sack , Phys. Med. Biol. 2007, 52, 675. 17228113 10.1088/0031-9155/52/3/010

[133] Z. Yin , J. D. Hughes , K. J. Glaser , A. Manduca , J. Van Gompel , M. J. Link , A. Romano , R. L. Ehman , J. Huston , J. Magn. Reson. Imaging 2017, 46, 1007. 28194925 10.1002/jmri.25623 PMC5600107

[134] K. Nael , E. Gibson , C. Yang , P. Ceccaldi , Y. Yoo , J. Das , A. Doshi , B. Georgescu , N. Janardhanan , B. Odry , M. Nadar , M. Bush , T. J. Re , S. Huwer , S. Josan , H. Von Busch , H. Meyer , D. Mendelson , B. P. Drayer , D. Comaniciu , Z. A. Fayad , Sci. Rep. 2021, 11, 6876. 33767226 10.1038/s41598-021-86022-7 PMC7994311

[135] B. Turkbey , M. A. Haider , Br. J. Radiol. 2022. 10.1259/bjr.20210563 PMC8978238 34860562

[136] R. Ahmed , M. J. Oborski , M. Hwang , F. S. Lieberman , J. M. Mountz , Cancer Manage. Res. 2014, 6, 149. 10.2147/CMAR.S54726 PMC3969256 24711712

[137] J. Zhang , J. J. Cunningham , J. S. Brown , R. A. Gatenby , Nat. Commun. 2017, 8, 1816. 29180633 10.1038/s41467-017-01968-5 PMC5703947

