# Supplementary material for: Changes in Tissue Fluidity Predict Tumor Aggressiveness In Vivo
Source: Adv Sci (Weinh). 2023 Aug 8;10(26):2303523. doi: 10.1002/advs.202303523 (PMC10502644; doi:10.1002/advs.202303523)
Supplement: Supplementary file 1 — Supporting Information [file ADVS-10-2303523-s005.pdf]

ADVANCED SCIENCE
Open Access

Supporting Information

for *Adv. Sci.*, DOI 10.1002/advs.202303523

Changes in Tissue Fluidity Predict Tumor Aggressiveness In Vivo

Frank Sauer, Steffen Grosser, Mehrgan Shahryari, Alexander Hayn, Jing Guo, Jürgen Braun, Susanne Briest, Benjamin Wolf, Bahriye Aktas, Lars-Christian Horn, Ingolf Sack and Josef A. Käs*

Extended Methods

Collagen gel preparation: The structure of these collagen gels is sensitive to variations in concentration in general and also pH and temperature during the polymerization process. For the preparation of 1 ml gel, 0.75 ml of rat tail collagen type 1 (4 mg/ml, Lot#190594, Serva, Heidelberg, Germany) or 0.75 ml bovine skin collagen type 1 (4 mg/ml, Lot#0235G, Biochrom, Berlin, Germany) were mixed with a phosphate buffer solution. The buffer solution was calculated individually for each gel type to neutralize the acidity of the collagen stock solution to a pH of 7.4 and consisted of 171 µl 1 M disodium hydrogen phosphate (Na_2HPO_4), 50 µl dH_2O and 29 µl 1 M sodium dihydrogen phosphate ($NaH_2PO_4{\cdot}H_2O$) [1]. The whole gel preparation process was done on ice to inhibit premature collagen polymerization. Immediately 500 µl of the activated gel solution was transferred into a 7 mm glass tube and put into an incubator for polymerization at 37 °C and 95 % humidity for 2 hours (rat-tail collagen) or 4 hours (bovine-skin collagen). Afterwards, the polymerized gels were rinsed three times with PBS and incubated overnight before use.

Tabletop MRE: The tabletop MRE setup consists of a compact MRI scanner (Pure Devices, Würzburg, Germany) with a 10 mm bore and a 0.5 T permanent magnet customized by an additional gradient amplifier (DC 600, Pure Devices, Würzburg, Germany). An MRI system-controlled piezoelectric driver (Piezosystem Jena, Jena, Germany) was mounted on top of the sample tube and driven by harmonic waveforms of frequencies between 200 and 6000 Hz. The collagen samples within the glass tubes (see [2] for additional details) were positioned within the bore of the MRI scanner, which was heated to 37 °C. The vibrations from the piezo actuator were constrained to purely axial motion direction and induced via the glass walls into the samples. A detailed overview of the imaging sequences and motion encoding gradients is described in [3]. In brief, the data acquisition time for each frequency was approximately 16 min, while a frequency range from 200 Hz to 2 kHz was covered in 100 Hz intervals. The following acquisition parameters were used: repetition time = 1000 ms, echo time = 42 ms, slice thickness = 3 mm, matrix size = 56 x 56, field of view = (8.4 x 8.4) mm² resulting in a voxel size of (0.15 x 0.15 x 3) mm³.

Confocal microscopy on collagen gels: Collagen gels were prepared as described above in a 24-well µ-Plate (ibidi, Martinsried, Germany) and stained overnight with TAMRA-SE (Sigma-Aldrich, Cat. No.: 21955), followed by three rinsing steps with PBS. Image stacks with 150 µm cubic dimension were recorded with a confocal laser scanning microscope (TCS SP8, Leica, Wetzlar, Germany) by using a 40x NA/1.10 water immersion objective under illumination of a 561 n, DPSS-Laser. 3D views were created using the Leica Application Suite X (3.1.5.16308, Leica, Wetzlar, Germany).

Confocal microscopy on primary human tumor explants: The analysis of primary human tumor tissue was approved by the ethics committees of the Medical Faculty of Leipzig University for mamma carcinoma (No. 073-13-11032013). Tumor samples were provided by the Institute of Pathology of Leipzig University Hospital direct after surgery. These samples were cut with a scalpel into millimeter-sized pieces and transferred into a 96-well µ-Plate

(ibidi, Martinsried, Germany) and cultured in standard cell culture medium (DMEM with 4.5 g/l glucose, L-glutamine supplemented with 10 % fetal bovine serum (Cat. No. A15-151, PAA) and 100 U/ml penicillin/streptomycin at 37 °C in a 5 % CO_2 atmosphere. Each 0.5 µM of SPY650-DNA and SPY555-Actin (Spirochrome) were added to the culture medium. Image stacks were acquired on a spinning disc confocal microscope (Axio Observer, Zeiss, Jena) every 10 min for 12 hours with a ZEISS Plan-Apochromat 25x/0.8 objective with water immersion under illumination from a 561 nm and from a 638 nm laser under incubation at 37 °C and 5 % CO_2. Background subtraction was performed with inbuilt Zeiss software, and for image registration, the plugin *Correct 3D Drift* [4] from FIJI [5] was used. Nucleus tracking was done manually with Matlab (version R2022a, Natick, MA, USA).

Extended Study data

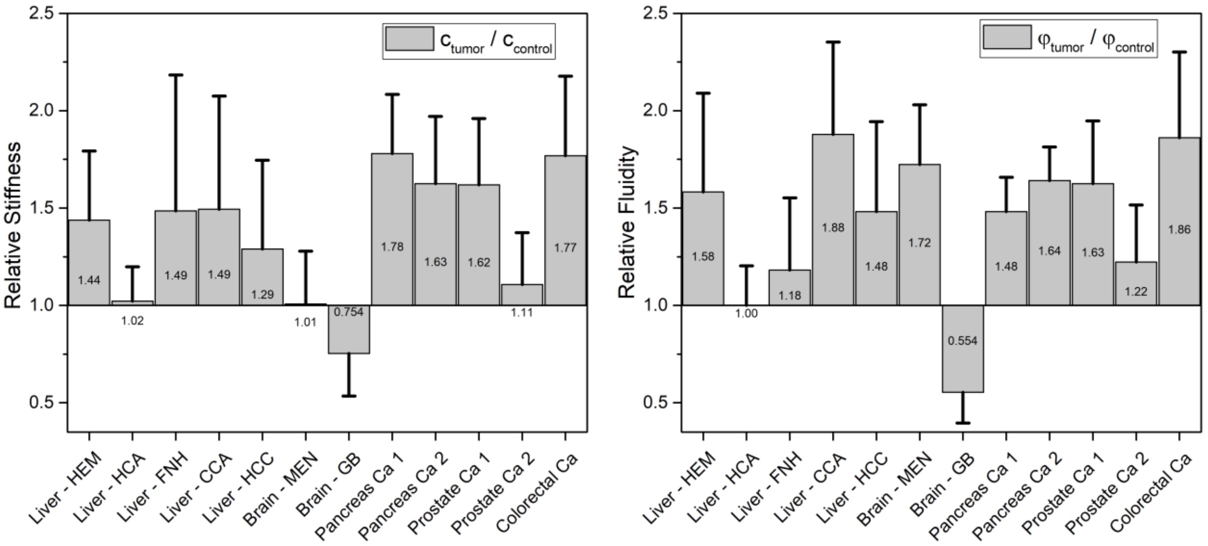

Supplementary figure F1: Relative Stiffness and Fluidity derived from the data shown in Tab.2. shows how the viscoelastic properties of tumors change when directly compared to their corresponding control tissue (for more details see Tab.2) as a baseline. The numeric values indicate the shear wave speed (c) ratio or the phase angle (φ) ratio tumor/control. Error show the standard deviations that were taken from the original publications (see also Tab.2) and are depicted only in one direction for improved visibility.

Primary human breast cancer sample used for cell tracking and supplemental videos

Patient information:
Age: 48 years
no prior diseases
BMI: 23.6

Preoperative pathology:
ductal invasive (NST - no special type). Grading: 1 (well differentiated).
Estrogen receptor expression: 100%
Progesterone receptor expression: 60%

HER2 negativ.
ki67: 2%
Overall: luminal A carcinoma.
Clinico-pathological stage: mcT2 pN0 cM0
Final pathology after surgery: ympT2 ypN1a (3/10) cM0 R0 G1 L1 V0 Pn0

m	multicentric
c	clinical examination, f.e. medical imaging
T2	primary Tumor stage 2 = largest dimension from 2 cm to 5 cm
p	after histopathological examination of surgical specimen
N0	no regional lymph nodes metastasis
M0	no metastases
y	after chemotherapy
N1a	metastases in axillary lymph nodes
R0	no residual tumor
G1	grade 1 highly differentiated
L1	invasion into lymphatic vessels
V0	no invasion into veins
Pn0	no perineural invasion

Estrogen receptor: 100%
Progesterone receptor: 85%
HER2 negative
ki67 2%

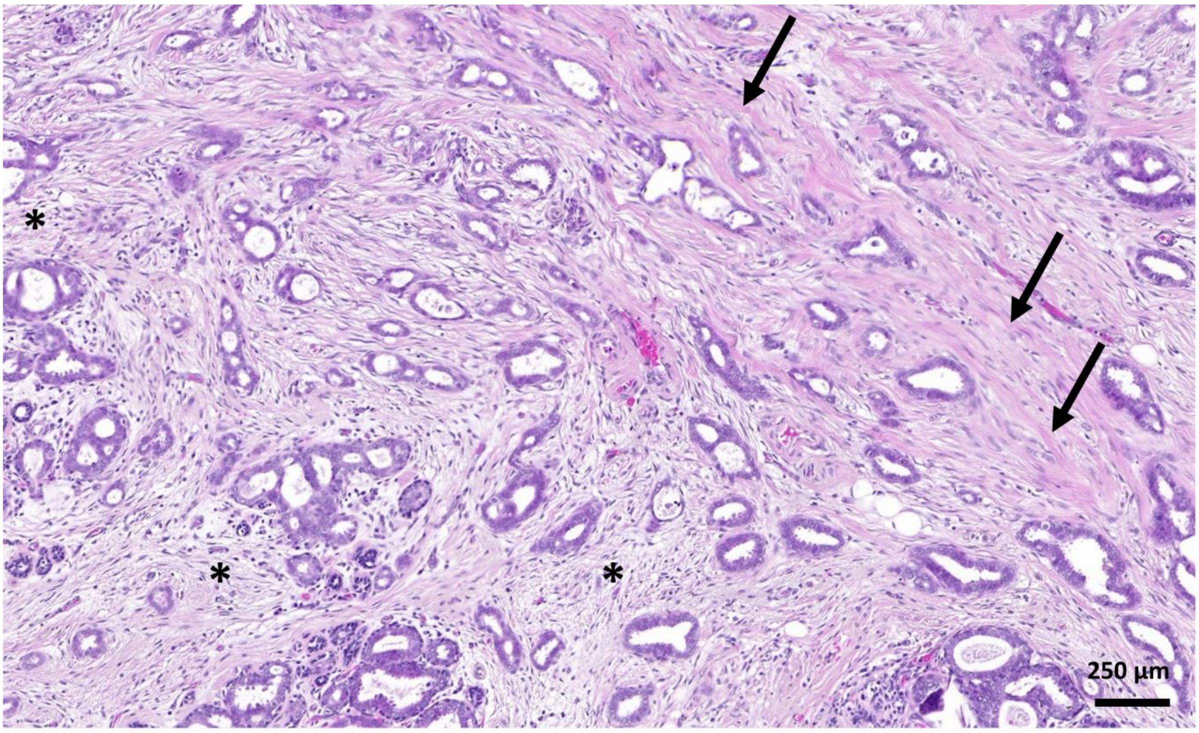

Supplementary figure F2: Additional H&E-stained histopathology slice from the same tumor as discussed in Fig.3 and mentioned above. Infiltrative breast cancer of no special type (NST; formerly known as invasive ductal carcinoma). Tumor cell clusters are surrounded by cancer associated fibroblasts (CAFs) embedded in stromal tissue. Exemplary CAFs are marked with asterisks (*) and

resting normal fibrotic tissue is exemplary marked with black arrows. Histopathological assessment done by senior pathologist.

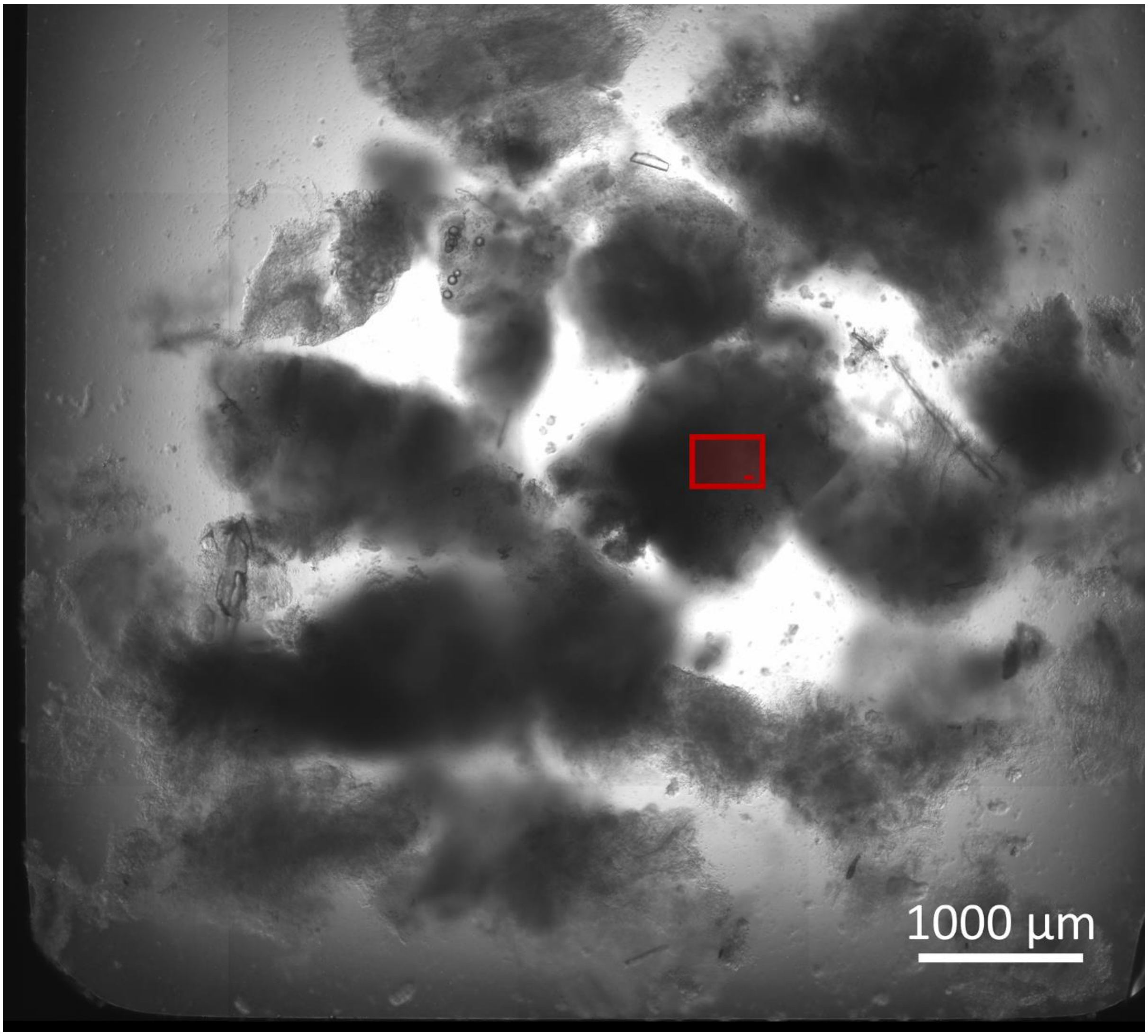

Supplementary figure F3: Brightfield image of millimeter-sized tissues pieces from the exact same tumor as shown in Fig.3a of the main manuscript and in supplementary figure F2. The tissue was cut manually with a scalpel and stained for cell nuclei and actin. This snapshot was taken immediately before starting the live measurement discussed in Fig3b & c. On this large scale, holes between the tissue pieces can be easily identified. Also, fatty tissue would be clearly visible due to its high refractive index. The supplemental videos S1 – S4 described below show live observations from exactly these tissue pieces. The small red insert represents the typical xy-dimensions of the videos. The short red line within equals 50 µm.

Supplemental videos:

S1_ClusterandStroma: Corresponding video to Fig. 4 a & b. Highly active, dense cell cluster with motile cells next to quasi-static stroma region of a primary human breast tumor explant. Manually dissected pieces of a tumor were stained with a vital DNA stain (red SPY650-DNA) and a vital F-actin stain (green, SPY555-Actin). Video created from 3D confocal images stacks recorded every 10 min over the course of 12 hours. The dimensions of the underlying image cube were (431 x 299 x 57) μm^3 with a 0.433 µm

resolution in x- and y-direction, while the distance in z between slices was 3 µm. To depict the volumetric nature of the image cubes, the scene is slightly rotated for better visualization.

S2_Acini: Highly active cells moving in acini-like structures of a primary human breast tumor explant. Manually dissected pieces of a tumor were stained with a vital DNA stain (red SPY650-DNA) and a vital F-actin stain (green, SPY555-Actin). Video created from 3D confocal images stacks recorded every 10 min over the course of 12 hours. The dimensions of the underlying image cube were (456 x 302 x 75) μm^3 with a 0.433 µm resolution in x- and y-direction, while the distance in z between slices was 3 µm. To depict the volumetric nature of the image cubes, the scene is slightly rotated for better visualization.

S3_ClusteronSubstrate: Cell cluster from primary human breast tumor explant dissolving on substrate indicating the viability of the cells. Actin Stress fibers are clearly visible for cells migrating on the substrate. Manually dissected pieces of a tumor were stained with a vital DNA stain (red SPY650-DNA) and a vital F-actin stain (green, SPY555-Actin). Video created from 3D confocal images stacks recorded every 10 min over the course of 12 hours. The dimensions of the underlying image cube were (425 x 305 x 57) μm^3 with a 0.433 µm resolution in x- and y-direction, while the distance in z between slices was 3 µm. To depict the volumetric nature of the image cubes, the scene is slightly rotated for better visualization.

S4_Stroma: Inactive or static cells in a stroma-like area within an explant of a primary human breast tumor. On the lower vessel structures can be identified. Manually dissected pieces of a tumor were stained with a vital DNA stain (red SPY650-DNA) and a vital F-actin stain (green, SPY555-Actin). Video created from 3D confocal images stacks recorded every 10 min over the course of 12 hours. The dimensions of the underlying image cube were (427 x 319 x 60) μm^3 with a 0.433 µm resolution in x- and y-direction, while the distance in z between slices was 3 µm. To depict the volumetric nature of the image cubes, the scene is slightly rotated for better visualization.

Detailed tumor classification scheme:

Tumor classification scheme (see also Fig. 4 main text) for all 72 parameter combinations:

Number	Tumor stiffer than control?	Fluidity tumor	Fluidity control	Heterogeneity cumor	Tumor front texture	Output Cases
1	no	solid	solid	no	sharp	1
2	no	solid	solid	yes	sharp	1
3	no	solid	solid	no	diffuse	3
4	no	solid	solid	yes	diffuse	3
5	no	solid	transitional	no	sharp	1
6	no	solid	transitional	yes	sharp	1
7	no	solid	transitional	no	diffuse	3
8	no	solid	transitional	yes	diffuse	3
9	no	solid	fluid	no	sharp	1
10	no	solid	fluid	yes	sharp	1
11	no	solid	fluid	no	diffuse	3
12	no	solid	fluid	yes	diffuse	3
13	no	transitional	solid	no	sharp	1
14	no	transitional	solid	yes	sharp	1
15	no	transitional	solid	no	diffuse	3

16	no	transitional	solid	yes	diffuse	3
17	no	transitional	transitional	no	sharp	1
18	no	transitional	transitional	yes	sharp	1
19	no	transitional	transitional	no	diffuse	3
20	no	transitional	transitional	yes	diffuse	3
21	no	transitional	fluid	no	sharp	1
22	no	transitional	fluid	yes	sharp	1
23	no	transitional	fluid	no	diffuse	3
24	no	transitional	fluid	yes	diffuse	3
25	no	fluid	solid	no	sharp	1
26	no	fluid	solid	yes	sharp	1
27	no	fluid	solid	no	diffuse	3
28	no	fluid	solid	yes	diffuse	3
29	no	fluid	transitional	no	sharp	1
30	no	fluid	transitional	yes	sharp	1
31	no	fluid	transitional	no	diffuse	3
32	no	fluid	transitional	yes	diffuse	3
33	no	fluid	fluid	no	sharp	1
34	no	fluid	fluid	yes	sharp	1
35	no	fluid	fluid	no	diffuse	3
36	no	fluid	fluid	yes	diffuse	3
37	yes	solid	solid	no	sharp	2
38	yes	solid	solid	yes	sharp	2
39	yes	solid	solid	no	diffuse	4
40	yes	solid	solid	yes	diffuse	4
41	yes	solid	transitional	no	sharp	2
42	yes	solid	transitional	yes	sharp	2 or 4
43	yes	solid	transitional	no	diffuse	4
44	yes	solid	transitional	yes	diffuse	4
45	yes	solid	fluid	no	sharp	2
46	yes	solid	fluid	yes	sharp	4
47	yes	solid	fluid	no	diffuse	4
48	yes	solid	fluid	yes	diffuse	4
49	yes	transitional	solid	no	sharp	2
50	yes	transitional	solid	yes	sharp	2 or 4
51	yes	transitional	solid	no	diffuse	4
52	yes	transitional	solid	yes	diffuse	4
53	yes	transitional	transitional	no	sharp	2 or 4
54	yes	transitional	transitional	yes	sharp	4
55	yes	transitional	transitional	no	diffuse	4
56	yes	transitional	transitional	yes	diffuse	4
57	yes	transitional	fluid	no	sharp	2 or 4
58	yes	transitional	fluid	yes	sharp	4
59	yes	transitional	fluid	no	diffuse	4
60	yes	transitional	fluid	yes	diffuse	4
61	yes	fluid	solid	no	sharp	2 or 4

62	yes	fluid	solid	yes	sharp	4
63	yes	fluid	solid	no	diffuse	4
64	yes	fluid	solid	yes	diffuse	4
65	yes	fluid	transitional	no	sharp	2 or 4
66	yes	fluid	transitional	yes	sharp	4
67	yes	fluid	transitional	no	diffuse	4
68	yes	fluid	transitional	yes	diffuse	4
69	yes	fluid	fluid	no	sharp	4
70	yes	fluid	fluid	yes	sharp	4
71	yes	fluid	fluid	no	diffuse	4
72	yes	fluid	fluid	yes	diffuse	4

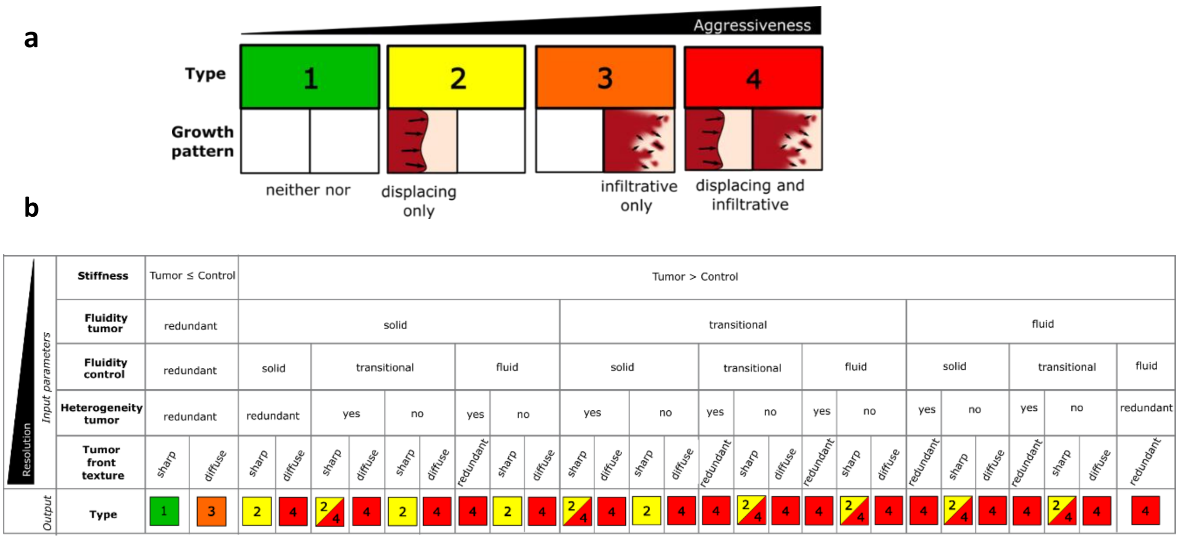

Supplementary figure F4: Classification scheme towards predictive tumor imaging based on tomoelastography. (a) Output types attributed to growth patterns and respective combinations. (b) Classification scheme derived from the gedankenexperiment: Course of action along the resolution axis from top to bottom for predictive tumor imaging based on the 5 input parameters and 4 output types presented in a). Tissues with transitional fluidity are considered in transition between fluid and solid material properties and can therefore have both traits. In those cases, the outcome is not always uniform and is depicted as type 2/4 . Redundant: not relevant for the outcome.

Necrosis in brain tumors (GB) shows higher fluidity and lower stiffness compared to healthy brain tissue

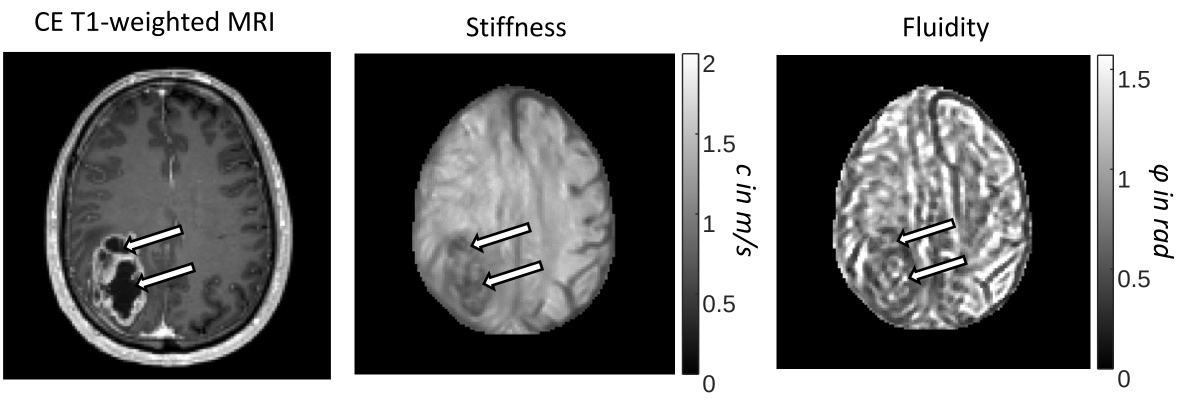

Supplementary figure F5 Tomoelastography results of a glioblastoma in a 68-year-old man. Shown are maps of stiffness (shear-wave speed c) and fluidity (phase angle φ) along with T1-weighted contrast enhanced (CE) MR images for anatomical orientation. Necrotic areas, can be identified by their typical heterogeneous high fluidity and simultaneously low stiffness (white arrow). Image taken from (6)

[1] C. T. Mierke, T. Fischer, S. Puder, T. Kunschmann, B. Soetje, and W. H. Ziegler, "Focal adhesion kinase activity is required for actomyosin contractility-based invasion of cells into dense 3D matrices," *Sci. Rep.*, vol. 7, no. 1, p. 42780, Feb. 2017.

[2] F. Sauer *et al.*, "Collagen networks determine viscoelastic properties of connective tissues yet do not hinder diffusion of the aqueous solvent," *Soft Matter*, vol. 15, no. 14, pp. 3055–3064, 2019.

[3] J. Braun *et al.*, "A compact 0.5 T MR elastography device and its application for studying viscoelasticity changes in biological tissues during progressive formalin fixation," *Magn. Reson. Med.*, vol. 79, no. 1, pp. 470–478, Jan. 2018.

[4] A. Parslow, A. Cardona, and R. J. Bryson-Richardson, "Sample drift correction following 4D confocal time-lapse imaging," *J. Vis. Exp.*, no. 86, Apr. 2014.

[5] J. Schindelin *et al.*, "Fiji: an open-source platform for biological-image analysis," *Nat. Methods*, vol. 9, no. 7, pp. 676–682, Jul. 2012.

6. Shahryari M. *et al.* Characterization of tumor mechanical properties in glioma patients using 3D phase-gradient inversion in multifrequency MR elastography. Joint Annual Meeting ISMRM-ESMRMB & ISMRT 31st Annual Meeting. 07-12 May 2022; London, England, UK. ISMRM (2022)
